# Prevalence and related factors of high myopia among adults aged 50 years and older in Fujian Eye Study

**DOI:** 10.1371/journal.pone.0355042

**Published:** 2026-08-03

**Authors:** Yang Li, Qinrui Hu, Bin Wang, Xiangdong Luo, Mingqin Zhang, Xiaoxin Li

**Affiliations:** 1 Eye Institute and Affiliated Xiamen Eye Center of Xiamen University, School of Medicine, Xiamen University, Xiamen, China; 2 Xiamen Clinical Research Center for Eye Diseases, Xiamen, Fujian, China; 3 Xiamen Key Laboratory of Ophthalmology, Xiamen, Fujian, China; 4 Translational Medicine Institute of Xiamen Eye Center of Xiamen University, Xiamen, Fujian, China; 5 Fujian Provincial Key Laboratory of Corneal & Ocular Surface Diseases, Xiamen, Fujian, China; 6 Xiamen Municipal Key Laboratory of Corneal & Ocular Surface Diseases, Xiamen, Fujian, China; 7 Department of Ophthalmology, Peking University People’s Hospital, Beijing, China; University of Missouri-Columbia, UNITED STATES OF AMERICA

## Abstract

**Purpose:**

This study aimed to assess the prevalence of high myopia and its associated demographic and ocular factors among residents aged 50 and older in Fujian Province, Southeast China.

**Methods:**

A population-based cross-sectional eye study was conducted from May 2018 to October 2019, enrolling residents aged 50 and older. Participants completed a questionnaire covering education, income, medical history, and lifestyle habits (including smoking, drinking, and tea consumption). They also underwent physical and ophthalmological examinations measuring height, weight, systolic blood pressure (SBP), diastolic blood pressure (DBP), heart rate (HR), refraction, intraocular pressure (IOP), and visual acuity (distance and best-corrected). Statistical analysis was performed using Stata software, and a multivariate logistic regression model was employed to identify factors associated with high myopia.

**Results:**

A total of 8,024 residents were included in the study. The overall prevalence of high myopia was 3.85% [95% CI: 3.43%−4.27%]. Multiple logistic regression showed that high myopia was significantly associated with inland residency (OR=0.672, p = 0.037), higher IOP (OR=1.069, p = 0.001) and higher education level (OR=1.645, p < 0.001).

**Conclusion:**

High myopia is prevalent among Chinese adults, affecting 3.85% of the study population. The findings highlight the need for greater investment in accessible eye care services and policies aimed at inland elderly residents, particularly those with higher IOP and higher education level.

## Introduction

Myopia, and especially high myopia, is recognized as a major public health concern. Myopia affected nearly 30% of the world population in 2020 and this number is expected to rise to 50% by 2050 [1]. Pathologic myopia is defined by the presence of typical complications in the fundus (posterior staphyloma or myopic maculopathy equal to or more serious than diffuse choroidal atrophy).Pathologic myopia often occurs in eyes with high myopia and is a major cause of visual impairment worldwide [2]. Although the prevalence of high myopia in young children is low, 10–20% of high school children in Asia have high myopia, with many still progressing, and one in three patients with high myopia develop visual impairment with age [3]. Myopia was associated with a lower prevalence of diabetic retinopathy, age-related macular degeneration and angle-closure glaucoma, while high myopia, more than moderate myopia, was associated with higher prevalence and incidence of open-angle glaucoma [4].

While adolescent myopia has dominated research agendas, epidemiological data on older adults remain strikingly limited. Adult-onset myopia is common, representing a third or more of all myopia in western populations, but less in East Asia, where onset during childhood is high. Clinically meaningful myopia progression continues in early adulthood and may average 1.00 diopters (D) between 20 and 30 years of age. Higher levels of myopia are associated with greater absolute risk of myopia-related ocular disease and visual impairment; thus, myopia in this age group requires ongoing management [5]. A review of 6555 reports found that the overall high myopia prevalence was 5.3%; projections for 2050 under minimum growth, experience-based, and maximum growth scenarios were 14.4%, 61.3%, and 71.9%, respectively [6]. The Raine Study showed the 8-year incidence of myopia and high myopia were 14.0% (95% CI, 11.5%−17.4%) and 0.7% (95% CI, 0.3%−1.2%), respectively [7]. A cross-sectional study among Han and Uyghur students in Xinjiang, China, showed that the overall age- and sex-adjusted prevalence of myopia and high myopia were 47.70% (95% CI: 47.67–47.74) and 2.55% (95% CI: 2.54–2.56), respectively [8]. Early-onset high myopia progresses to pathological forms in later life and demands urgent investigation into aging cohorts [9].

Crucially, diagnostic standardization remains contentious. Although axial length measurement enhances pathological myopia identification, large-scale surveys frequently adopt SE ≤−6.00 D as a pragmatic threshold, balancing accuracy with feasibility—an approach endorsed by the International Myopia Institute (IMI) for population screening [10]. This operational definition underpins our study, which aims to address several critical gaps in the current literature. First, there remains a scarcity of population-based data on high myopia specifically among the elderly in China. Second, evidence regarding modifiable risk factors beyond genetic predisposition is still limited. Third, regional healthcare disparities within Fujian Province — where notable coastal-inland socioeconomic gradients exist — may influence ocular health outcomes, yet this has been underexplored. By investigating the prevalence patterns and associated factors in this aging cohort, our findings aim to inform targeted vision preservation strategies and contribute to the ongoing global discussion on defining and assessing myopia in epidemiological contexts.

## Materials and methods

### Study design

The Fujian Eye Study (FJES) employed a multistage random cluster sampling design. Clusters were defined as administrative villages/urban communities, stratified by coastal-inland geography and urbanization level (urban/rural) based on 2017 census data [11]. Of 120 selected clusters, 10,044 eligible residents were invited, with 8,211 participating (response rate: 81.7%). Non-participants did not differ significantly in age or sex distribution (p = 0.12).

### Ethics approval and consent to participate

A clinical study registry was obtained for the 2018–2019 FJES study (Clinical trial number: ChiCTR2100043349, registration date: 2021-02-21) and the study protocol was approved by the Ethics Committee of Xiamen Eye Center affiliated with Xiamen University (Acceptance No. XMYKZX-KY-2018–001).

### On-site examination

The report encompasses the following key components: a questionnaire addressing factors such as age, sex, level of urbanization, geographic location, educational background, income, phone usage habits (including phone use in dark environments), history of chronic diseases, and consumption of alcohol, tobacco, and tea. Additionally, it includes measurements of height, weight, body mass index (BMI), systolic blood pressure (SBP), diastolic blood pressure (DBP), heart rate (HR), intraocular pressure (IOP), presenting distance visual acuity (PDVA), best-corrected visual acuity (BCVA), and refractive status.

The variable “phone use in dark environments” was based on participant self-report in our questionnaire: “Do you use mobile phone after turning off the lights at night?” Responses were recorded as a binary variable (Yes/No). This measure was intended to capture a common behavioral pattern rather than a precisely quantified photometric state.

An experienced technician measured participants’ IOP with the handheld iCare rebound tonometer (RBT; Icare TA 01, Finland). Quintic measurements were obtained; measurements with poor signal quality (indicated by the device) were automatically discarded and not included in the set of five valid readings required for the average, and their mean was recorded and taken for further statistical analysis. The RBT has demonstrated good intra-operator repeatability in field studies. In order to improve reliability, our study employed the same operator for all measurements. In randomized controlled and observational studies, the iCare rebound tonometer has been validated against the Goldmann flattening tonometer, showing good correlation and reproducibility [12–14].

### Refractive assessment protocol

Objective refraction parameters — including spherical power (DS), cylindrical power (DC), and axis orientation — were quantified using the Topcon KR800 autorefractor (Topcon Corporation, Tokyo, Japan). The Topcon KR800 autorefractor is a validated instrument with high accuracy and repeatability for population-based studies [15]. Three consecutive measurements per eye were obtained, with the arithmetic mean adopted as the definitive refractive value to minimize intraoperator variability. If a measurement was deemed unstable or an error message appeared, the technician would retake it. The arithmetic mean was calculated from three consecutive valid measurements. Visual function evaluation incorporated monocular presentation distance visual acuity (PDVA), assessed at 5 meters using the E-chart, and best-corrected visual acuity (BCVA), determined by retesting with optimal spherocylindrical correction refined through a trial frame. Based on the calculated spherical equivalent (SE = spherical power + cylindrical power/2), participants were classified as having high myopia if SE was ≤ −6.00 diopters [16].

### Statistical methodology

Data processing and analyses were executed using Stata/SE v15.1 (StataCorp LLC, TX, USA). Categorical variables were initially assessed using Pearson’s χ² test with Yates’ continuity correction where appropriate.

Univariate logistic regression models were first constructed to screen potential risk factors related to high myopia, with effect sizes expressed as odds ratios (ORs) and 95% confidence intervals (CIs). Multivariable analysis subsequently employed generalized linear mixed models incorporating cluster-robust standard errors to account for intra-cluster correlations inherent in the sampling design. Statistical significance was defined a priori at two-tailed p < 0.05, with all estimates reported to three decimal places to enhance precision.

## Results

### Characteristics of the participants

A total of 8,211 residents aged 50 and older were surveyed, and after excluding those with incomplete data, 8,024 individuals were included in the study. Among the participants, 309 (3.85% [95% CI: 3.43%−4.27%]) were identified as having high myopia. Of these, 179 (57.9%) were female, 177 (57.3%) resided in urban areas, and 222 (71.8%) lived in coastal regions. The prevalence of high myopia across the primary subgroups is summarized in Table 1.

**Table 1:** The characteristics of the study participants and the prevalence of high myopia (n = 8024).

Characteristics		Total population	High myopia	Prevalence (%)	P-value
Age (years) group	50–54	1139	89	7.25	<0.001
	55–59	1323	41	3.01	
	60–64	1529	41	2.61	
	65–69	1583	59	3.59	
	70–74	1126	41	3.51	
	75–79	560	26	4.44	
	80 and above	455	12	2.57	
	Total	8024	309	3.85	
Sex	Male	3273	130	3.97	0.640
	Female	4751	179	3.77	
	Total	8024	309	3.85	
Urbanization	Urban	4583	177	3.86	0.952
	Rural	3441	132	3.84	
	Total	8024	309	3.85	
Geographic location	Coastal	6303	222	3.52	0.003
	Inland	1721	87	5.06	
	Total	8024	309	3.85	
Education level	Illiteracy	1242	34	2.74	<0.001
	Primary school	1487	45	3.03	
	Middle school	3039	92	3.03	
	College and above	1178	102	8.66	
	Total	6946	273	3.93	
Income level	<=2000	2352	69	2.93	<0.001
	2000-5000	1918	83	4.33	
	>5000	579	41	7.08	
	Total	4849	193	3.98	
Duration of phone use (hours, h)	0	2136	70	3.28	0.003
	< 1	2631	93	3.53	
	1–2	1030	45	4.37	
	>2	1005	59	5.87	
	Total	6802	267	3.93	

### Correlations between high myopia and sociodemographic characteristics

The associations between various demographic, socioeconomic, lifestyle factors and high myopia were first assessed using univariate logistic regression. In the overall study population, high myopia (OR = 0.686, p = 0.004) showed a significant association with geographic location, with a notably lower prevalence observed in coastal areas compared to inland regions. Conversely, high myopia (OR = 1.007, p = 0.952) demonstrated no significant correlation with urbanization, whether comparing urban areas to rural ones.

High myopia was found to be strongly associated with higher education levels (OR = 1.549, p < 0.001) and higher income (OR = 1.576, p < 0.001). As education and income levels increased, the prevalence of high myopia correspondingly rose. Furthermore, individuals who frequently used their phones exhibited a higher prevalence of high myopia (OR = 1.229, p < 0.001), with the rate increasing in relation to prolonged phone usage. The prevalence was notably higher among those who used their phones in dark environments (OR = 1.415, p = 0.008) compared to those who did not.

### Correlations between high myopia and biological characteristics

In general, high myopia was strongly associated with younger age (OR = 0.976, P < 0.001), with its highest prevalence occurring among individuals aged 50–54 years. After the age of 55, the prevalence of high myopia appeared to stabilize. No significant associations were found between high myopia and sex (OR = 0.947, p = 0.640), tobacco use (OR = 0.909, p = 0.565), alcohol consumption (OR = 1.069, p = 0.686), or tea consumption (OR = 1.002, p = 0.989).

Height showed a significant association with high myopia (OR = 1.018, p = 0.015), with its prevalence rising alongside an increase in height. However, no significant correlation was observed with weight (OR = 1.003, p = 0.606) or BMI (OR = 0.984, p = 0.382).

High myopia showed no correlation with SBP (OR = 1.000, p = 0.985), DBP (OR = 1.001, p = 0.756), or HR (OR = 1.009, p = 0.069). Additionally, it was found to be independent of any history of chronic diseases, including hypertension (OR = 0.963, p = 0.745), diabetes (OR = 0.897, p = 0.408), and hyperlipidemia (OR = 1.118, p = 0.481).

A strong correlation was identified between high myopia and elevated IOP (OR = 1.085, p < 0.001), with the prevalence of high myopia rising in tandem with increasing IOP levels.The results of univariate logistic regression analyses for all examined factors are presented in Table 2.

**Table 2:** The univariate logistic regression of high myopia among each subgroups (n = 8024).

Characteristics	Odds Ratio	95% Confidence Interval (CI)	P-value
Age (years)	0.976	0.963 to 0.989	<0.001
Sex	0.947	0.752 to 1.192	0.640
Urbanization	1.007	0.800 to 1.267	0.952
Geographic location	0.686	0.532 to 0.884	0.004
Height (cm)	1.018	1.003 to 1.033	0.015
Weight (kg)	1.003	0.992 to 1.014	0.606
Body Mass Index (BMI)	0.984	0.950 to 1.020	0.382
Systolic blood pressure (SBP)	1.000	0.995 to 1.005	0.985
Diastolic blood pressure (DBP)	1.001	0.992 to 1.011	0.756
Heart rate (HR)	1.009	0.999 to 1.020	0.069
Diabeties Millitus (DM)	0.897	0.694 to 1.160	0.408
hypertension	0.963	0.766 to 1.210	0.745
Hyperlipidemia	1.118	0.820 to 1.523	0.481
Education level	1.549	1.349 to 1.778	<0.001
Income level	1.576	1.293 to 1.921	<0.001
Intraocular pressure (IOP)	1.075	1.045 to 1.107	<0.001
Phone use in dark environments	1.415	1.095 to 1.828	0.008
Phone use	1.301	0.986 to 1.717	0.063
Duration of phone use groups	1.229	1.095 to 1.379	<0.001
Smoking	0.909	0.655 to 1.260	0.565
Alcohol consumption	1.069	0.773 to 1.479	0.686
Tea consumption	1.002	0.777 to 1.292	0.989

### Multiple logistic regression

A multiple logistic regression analysis was used to evaluate various parameters, including age, geographic location (coastal vs. inland), height, IOP, education level, income, phone usage duration, and phone use in darkness. The findings, summarized in Table 3, revealed that after adjustments, high myopia was significantly associated with inland residency, elevated IOP and higher education level. In contrast, age, height, income, phone usage duration, and phone use in darkness no longer emerged as significant predictors.

**Table 3:** The multiple logistic regression in high myopia with several research factors in Fujian Eye Study.

Research factors	High myopia		
	Odds Ratio	95% Confidential Interval (CI)	*P*-value
Geographic location	0.672	0.462 to 0.977	0.037
Age	0.984	0.965 to 1.003	0.105
Height	0.985	0.964 to 1.006	0.151
IOP	1.069	1.027 to 1.114	0.001
Education	1.645	1.289 to 2.100	<0.001
Income	1.067	0.815 to 1.398	0.636
Phone use in dark environments	0.911	0.644 to 1.290	0.599
Duration of phone use groups	1.010	0.926 to 1.306	0.279

## Discussion

It is projected that by 2050, 9.8% of the population worldwide will have high myopia [17,18]. The development of high myopia is largely influenced by a combination of genetic, environmental, behavioral, and other contributing factors. This cross-sectional eye study examined the prevalence of high myopia and its related factors, including biological and sociodemographic variables, among adults aged ≥ 50 years in Southern China. The analysis explored a broad range of potential contributing elements, such as age, sex, urbanization, geographic location, education level, income, height, weight, SBP, DBP, IOP, and medical histories of diabetes, hypertension, or hyperlipidemia. Additionally, lifestyle habits like phone use duration, phone use in the dark, and the consumption of tobacco, alcohol, or tea were also investigated. In this study, we identified significant correlations between high myopia and living in inland areas, higher educational attainment, and elevated intraocular pressure.

### The prevalence of high myopia

The IMI has recommended defining high myopia as a spherical equivalent refractive error of ≤ −6.00 D when ocular accommodation is relaxed [10]. In alignment with IMI’s guidelines, our study also adopted the threshold of −6.00 D or less to classify high myopia. Consistency in research standards is essential for ensuring comparability in big data analyses and for yielding more robust and convincing findings in future studies. A systematic review and meta-analysis of 145 articles revealed that only 59 studies explicitly defined and measured high myopia. Among these, 30.5% defined high myopia as −6.00 D or less, another 30.5% as less than −6.00 D, 35.6% as −5.00 D or less, while 1.7% defined it as −8.00 D or less, and an additional 1.7% as −3.00 D or less [18].

The prevalence of high myopia varies widely due to various limiting factors, including differences in countries, regions, age ranges, sex, and definitions [4–16]. Moreover, there are relatively few reports addressing high myopia among the elderly population [19–23]. Our study found that the prevalence of high myopia among adults aged ≥ 50 years in southeast China was 3.85%. One segment of the CNHS study involving Yi and Han adults aged from 40 to 80 years in Yunnan reported a prevalence of high myopia of 2.64% (95% CI, 1.75–3.53%) [19]. In another part of the CNHS, conducted among Mongolian and Han adults aged from 40 to 80 years in Inner Mongolia, the prevalence of high myopia (SE < −6.0D) was recorded at 3.6% (95% CI, 2.8–4.4%) [20]. Similarly, a cross-sectional study of Yugur and Han adults aged from 40 to 80 years living in Gansu, Northwest China, reported a prevalence of high myopia (SE < −6.0D) of 3.6% [21]. In eastern China, a community-based cross-sectional survey of residents aged ≥ 60 years in a rural setting revealed that the age-adjusted prevalence of high myopia was 2.5% (95% CI, 2.1–2.9) [22]. In comparison, the Tajimi Study (residents aged ≥ 40 years) showed that the prevalence of high myopia (SE <−5.0 diopters) was noticeably higher at 8.2% (95% CI, 7.2–9.2%) [23].

### Factors linked to high myopia

Two decades ago, multiple reports indicated that urban residents, individuals with higher education levels, and computer users were more prone to myopia, with the condition also being associated with higher intelligence [24–28]. The CNHS in Han and Yi populations aged from 40 to 80 years in Yunnan revealed that the Yi population had a lower prevalence of high myopia compared to the Han population (1.31% vs 3.34%, p = 0.049). In that study, myopia was found to be associated with ethnicity, age, height, time spent in rural areas, higher education level, and diabetes [19]. Similar results were observed in another CNHS analysis involving Han and Mongolian populations aged 40–80 in Inner Mongolia [20]. Nonetheless, our study showed that high myopia was independently associated with inland residency and higher education, but not with age, height, urbanization, income, or prolonged phone use. This may be attributed to multiple interacting mechanisms: In aging populations, nuclear sclerosis-induced hyperopic shift may counterbalance pre-existing myopic progression, particularly in early-onset high myopia. This biomechanical equilibrium could obscure age-dependent trends, as observed in the Beijing Eye Study [29]. While taller stature correlates with longer axial length in adolescents, this relationship attenuates in older adults due to age-related scleral remodeling and vitreous degeneration. Our findings align with the Singapore Longitudinal Aging Study, which showed null height-myopia associations after 60 years of age [30]. All these interpretations underscore the necessity of lifespan myopia trajectory analyses and refined exposure metrics in aging populations.

The JPHC-NEXT Eye Study investigated adults (age ≥ 40 years) without ocular surgery history and found that the prevalence of high myopia was 3.8% in males and 5.9% in females, revealing a significant difference [31]. However, our study reported no significant sex-related differences. While age was inversely associated and IOP positively associated, no correlations were observed between high myopia and other factors, including corneal structure, corneal endothelial cell density, height, BMI, HR, BP, biochemical profile, or current systemic and ocular disorders in either sex [31]. Furthermore, our findings indicated the association between high myopia and IOP, but no association with age. This may be related to axial myopia and scleral thinning, or measurement errors such as the influence of corneal thickness on IOP measurement [32–34]. As a cross-sectional study, our findings can demonstrate a statistical association but cannot establish causality or determine the direction of the relationship. Recent research showed anatomically,thinner trabecular meshwork increases the risk of high IOP, conversely lamina cribrosa defects may offer a fluid outlet, potentially mitigating the pressure [33]. While another review showed that scleral matrix remodeling has been shown to contribute to the biomechanical susceptibility of the sclera to accommodation-induced IOP fluctuations, resulting in reduced scleral thickness, axial length (AL) elongation, and axial myopia [34]. Thus, longitudinal or interventional studies are needed to clarify any causal relationship in the future.

The cross-sectional NHNES study suggested that myopia is linked to higher education levels in the U.S. [29], aligning with our findings. In contrast, both the Beijing Eye Study and the Central India Eye and Medical Study indicated no significant difference or even lower educational levels among the highly myopic group compared to the non-highly myopic group [35]. The inconsistent associations between educational attainment and high myopia across studies may stem from multifaceted interactions of methodological, demographic, and biological factors, such as the regional difference, the racial difference, gene-environment interactions, and unequal access to vision care. All these findings highlight the mutual influence of multiple factors on the correlation between education and myopia, reminding us that future research needs to comprehensively investigate multiple factors and more accurately reveal the correlation between diseases and factors.

### Limitations

This investigation has several constraints. First, the cross-sectional design inherently restricts causal inference between identified risk factors and high myopia, as temporal relationships cannot be established. Second, the absence of axial length measurements—a gold-standard biomarker for pathological myopia—limits our ability to differentiate physiological high myopia from sight-threatening pathological forms, potentially obscuring clinical risk stratification. Third, this study lies in its focus on the prevalence of high myopia as defined, along with an analysis of related factors such as social demographics and systemic diseases. However, it does not include a correlation analysis of eye diseases, for example, severe high myopia patients with early-onset complications (e.g., retinal detachment) may have been excluded from this community-based sample due to mobility limitations or prior hospitalization, truncating the observable risk spectrum, which we intend to address as the focal point of our future research. Fourth, the data for this study were collected between 2018 and 2019. While the prevalence of high myopia in older adults is unlikely to change dramatically within a 1–2 year period, secular trends in environmental factors (e.g., digital device use) among preceding generations could influence future estimates. Nevertheless, the identified associations between high myopia and factors like education and IOP are likely to remain relevant, and this study provides a crucial baseline for this aging cohort in Southern China.

In summary, this study is the first to report the prevalence of high myopia among adults (age ≥ 50 years) in Fujian Province, China. The prevalence rate was found to be 3.85%, and it was associated with inland residency, higher levels of education, and elevated IOP. We recommend integrating axial length measurements in future surveys and prioritizing vision screening for inland populations with limited healthcare access. Notably, the prevalence and contributing factors of high myopia differ across other racial groups and regions of China, potentially reflecting variations in environmental conditions, lifestyles, and genetic influences related to refractive errors. This research provides valuable epidemiological data to support regional policy-making efforts. To better understand the detailed associations between geographic location, IOP, educational background, and high myopia, further studies are needed across diverse age groups and ethnic populations, with a particular emphasis on investigating the underlying mechanisms.

## Supporting information

S1 File PLOSOne human subjects research checklist. (DOCX)

S2 File HM dataset. (XLSX)
