# Supplementary material for: Prevalence and related factors of high myopia among adults aged 50 years and older in Fujian Eye Study
Source: PLoS One. 2026 Aug 3;21(8):e0355042. doi: 10.1371/journal.pone.0355042 (PMC13432112; doi:10.1371/journal.pone.0355042)
Supplement: Attachment — Submitted filename: REVIEW 1 - PONE-D-25-14251.docx [file pone.0355042.s003.docx]

**REVIEW - PONE-D-25-14251**

**1. Summary of the research**

The study aimed to report the prevalence of high myopia in an older adult and elderly/aged population, as well as the factors associated with high myopia. This was to provide evidence that was lacking on high myopia among aged populations who are at higher risk of complications of high myopia. The authors report a prevalence of 3.85% in their population, with high myopia being significantly correlated with higher IOP and higher educational level. The authors conclude with a recommendation for increased eye care accessibility among the aged, especially those with higher IOP and educational attainment levels.

This research provides evidence on the prevalence and associated risk factors for high myopia in an aged population who are at higher risk for high myopia-associated ocular complications. This was previously lacking as previous literature mostly focused on younger populations.

A major strength of the study is related to the sampling: large sample size, high response rate (>80%) and report of non-significant variation between respondents and non-respondents of the study. However, a major limitation of this study is the time-sensitivity of the findings. The data was collected in 2018-2019, putting the study at risk of outdated data and questioning how representative the findings are of the current Fujian population. Overall, this was an important study that was well executed. I recommend that the authors address some minor issues and effect necessary revisions of the current manuscript version. Specific comments are below:

**2. Examples and evidence**

***Major issues***

N/A

***Minor issues***

Abstract:

1. Authors mention “blood type”. However, per the general understanding of blood type (as blood group), there are no records of data collection on blood type in the methods section, and no records of it in the results section as well. Can authors clarify what they mean by blood type and exclude it from the abstract to avoid confusion.

Introduction:

2. On page 3, paragraph 2, references 10 and 11 cited do not appear to be supportive of claim stated as authors suggest. Reference 10 talks about myopia in Europe, whereas reference 11 is on primary open-angle glaucoma with no data on myopia. Authors should re-check their references.

Materials and Methods:

3. Page 5, subsection “On-site examination”: What was the repeatability of the technician's measurement of IOP? How was intra-operator variability assessed?
4. Page 5, subsection “On-site examination”: How was the accuracy of the tonometer determined and how accurate was it? How repeatable were its readings?
5. Page 5, subsection “On-site examination”: Were outliers included/excluded in the calculation of mean IOP measurements or were they replaced with additional measurements?
6. Page 5, subsection “Refractive error assessment protocol”: How was the accuracy of the autorefractor determined and how accurate was it? How repeatable were its readings?
7. Page 5, subsection “Refractive error assessment protocol”: Were outliers included/excluded in the calculation of the arithmetic mean refractive error or were they replaced with additional measurements?
8. Page 5, subsection “Refractive error assessment protocol”: Who and how many people did the trial frame refinement? If many, how was the inter-operator variability tested and what was it?

Results:

9. On page 7-8, under the subsections “Correlations Between High Myopia and Sociodemographic Characteristics” and “Correlations Between High Myopia and Biological Characteristics”, I recommend that authors present the full tabulated results for the correlation analyses (univariate logistic regression analyses).

Discussion:

10. Page 10, paragraph 1, last line: The authors' results section seem to suggest the opposite of the claim stated here. From Table 1 and 2, higher educational level is reported to be significantly correlated with high myopia. However, the phrase “lower educational attainment” is contradictory. Can the authors clarify this?
11. Page 11, subsection “Factors Linked to High Myopia”, first sentence: Reference 24 and 26 are a duplication of the same article.
12. Page 11, subsection “Factors Linked to High Myopia”: What is the alignment between the Yugur and Han populations study and the authors' current study, considering that the authors did not assess family history of myopia, and did not find any association between high myopia and urbanization/rural-living? The only similarity between the two studies, as I can tell, is in relation to aging, which was later found not to be correlated with high myopia after adjustments in the multiple logistic regression analyses. I suggest that authors rephrase this to avoid confusion and propagating misleading information.
13. Page 11, subsection “Factors Linked to High Myopia”: What do the authors mean by certain features of educational relevance?
14. Page 12, subsection “Factors Linked to High Myopia”: The statement “*Severe high myopia patients with early-onset complications (e.g., retinal detachment) may have been excluded from this community-based sample due to mobility limitations or prior hospitalization, truncating the observable risk spectrum*” appears to be a limitation of the study, and therefore, is misplaced, disrupting the flow of the manuscript. Authors should consider restructuring to enhance clarity and flow.
15. Page 12, subsection “Factors Linked to High Myopia”: The sentence “*The cross-sectional NHNES study suggested that myopia is linked to higher education levels in the U.S.,[29] aligning with our findings.*” See comment 10.
16. Page 12, subsection “Factors Linked to High Myopia”: References 30-35 need reformatting. References 34 and 35 appear to have been included later, and so it has been numbered wrongly. Authors should reformat references to ensure appropriate and orderly numbering.
17. Page 13, “*All these findings highlight context-dependent "environmental sensitivity thresholds" shaping education-myopia relationships, remind us that future research needs to comprehensively focus on the impact of multiple factors*”: This statement has grammatical issues. Authors should consider rephrasing to fix grammar and enhance clarity.
18. ROC curves: Authors do not discuss figures 1 and 2 which are the ROC curves. I recommend that authors discuss what the curve means and their implications in terms of their findings.

Limitations:

19. Considering the time of the data collection, authors should address issues relating to the time-sensitivity of the results, and potential implication of outdated data.
