# Supplementary material for: Prevalence and related factors of high myopia among adults aged 50 years and older in Fujian Eye Study
Source: PLoS One. 2026 Aug 3;21(8):e0355042. doi: 10.1371/journal.pone.0355042 (PMC13432112; doi:10.1371/journal.pone.0355042)
Supplement: Attachment — Submitted filename: renamed_595bf.docx [file pone.0355042.s004.docx]

Prevalence and related factors of high myopia among adults aged 50 years and older in Fujian Eye Study

Yang Li ^1,2,3,6^, Bin Wang ^1,2,3,4,5,6^, Xiangdong Luo ^1,2^, Mingqin Zhang ^1,2^, Qinrui Hu ^1,2,3,6*^, Xiaoxin Li ^1,2,3,6,7*^

^1^Eye Institute and Affiliated Xiamen Eye Center of Xiamen University, School of Medicine, Xiamen University, Xiamen, China.

^2^Xiamen Clinical Research Center for Eye Diseases , Xiamen, Fujian, China.

^3^Xiamen Key Laboratory of Ophthalmology，Xiamen, Fujian, China.

^4^Fujian Provincial Key Laboratory of Corneal & Ocular Surface Diseases, Xiamen, Fujian, China.

^5^Xiamen Municipal Key Laboratory of Corneal & Ocular Surface Diseases, Xiamen, Fujian, China.

^6^Translational Medicine Institute of Xiamen Eye Center of Xiamen University, Xiamen, Fujian,  China.

^7^Department of Ophthalmology, Peking University People’s Hospital, Beijing100044, China.

^*^Corresponding authors:

Xiaoxin Li, MD, PhD, Xiamen University Xiamen Eye Center, Xiahe Road 336, Siming District, Xiamen, Fujian361003, China. Peking University People’s Hospital, Beijing100044, China. E-mail: [drlixiaoxin@163.com](mailto:drlixiaoxin@163.com)

Qinrui Hu, PhD, Xiamen University Xiamen Eye Center, Xiahe Road 336, Siming District, Xiamen, Fujian361003, China. E-mail: [huqinrui145@sina.com](mailto:huqinrui145@sina.com)

Running head: High myopia in Fujian Eye Study

Abstract

Purpose:
This study aimed to assess the prevalence of high myopia and its associated demographic and ocular factors among residents aged 50 and older in Fujian Province, Southeast China.

Methods:
A population-based cross-sectional eye study was conducted from May 2018 to October 2019, enrolling residents aged 50 and older. Participants completed a questionnaire covering education, income, blood type, medical history, medication use, lifestyle habits (including smoking, drinking, and tea consumption). They also underwent physical and ophthalmological examinations measuring height, weight, systolic blood pressure (SBP), diastolic blood pressure (DBP), heart rate (HR), refraction, intraocular pressure (IOP), and visual acuity (distance and best-corrected). Statistical analysis was performed using Stata software, and a multivariate logistic regression model was employed to identify factors associated with high myopia. The ROC curve was used to assess predictive accuracy.

Results:
A total of 8,024 residents were included in the study. The overall prevalence of high myopia was 3.85% [95% CI: 3.43%-4.27%]. Multiple logistic regression showed that high myopia was significantly associated with higher IOP (OR=1.07, p=0.001) and higher education levels (OR=1.71, p<0.001).

Conclusion:
High myopia is prevalent among Chinese adults, affecting 3.85% of eyes in the study population. The findings highlight the need for greater investment in accessible eye care services and policies aimed at inland residency elderly, particularly those with higher IOP and higher education levels.

**Keywords:** high myopia, cross-sectional eye study, adult, prevalence, related factor

Introduction

Myopia has emerged as a global public health challenge, with uncorrected refractive errors accounting for 53% of all vision impairment cases worldwide.^[1]^ Among its severe manifestations, high myopia—defined by the American Academy of Ophthalmology (AAO) as spherical equivalent (SE) ≤−6.00 diopters (D) combined with axial length ≥26.5 mm—predisposes individuals to vision-threatening complications including myopic maculopathy, retinal detachment, and glaucoma.^[2-3]^ Notably, myopic macular degeneration has surpassed diabetic retinopathy as the leading cause of irreversible blindness in China and Europe, underscoring its growing socioeconomic burden.^[4-6]^

While adolescent myopia has dominated research agendas, epidemiological data on older adults remain strikingly limited. A meta-analysis of 145 global studies revealed that merely 12% included participants aged ≥50 years, despite projections indicating that high myopia prevalence will escalate to 9.8% by 2050.^[7,8]^ This oversight is particularly concerning for East Asia, where population aging coincides with exceptionally high myopia rates: 80-90% among young adults versus 3.6-8.2% in those over 40.^[9-11]^ The "myopia time bomb" phenomenon—where early-onset high myopia progresses to pathological forms in later life—demands urgent investigation into aging cohorts.^[12]^

China exemplifies this dual burden. Urban myopia prevalence surged from 22.9% to 31.5% among adults ≥40 between 2001-2015, paralleling Japan's rise from 37.7% to 45.8% (2005-2017).^[13-14]^ European data further suggest a 5.7% generational increase in myopia among 50-79 year-olds.^[15]^ However, significant disparities persist: African populations exhibit <10% prevalence versus Iran's 1.9% for SE ≤−6.00 D, highlighting roles of genetic and environmental factors.^[8,16]^

Crucially, diagnostic standardization remains contentious. Although axial length measurement enhances pathological myopia identification, large-scale surveys frequently adopt SE ≤−6.00 D as a pragmatic threshold, balancing accuracy with feasibility—an approach endorsed by the International Myopia Institute (IMI) for population screening.^[17]^ This operational definition underpins our study, which addresses three critical gaps:

1.Scarcity of data on high myopia among Chinese elderly;

2.Limited evidence regarding modifiable risk factors beyond genetic predisposition;

3.Regional healthcare disparities in Fujian Province, where coastal-inland socioeconomic gradients may influence outcomes.

By investigating prevalence patterns and associated factors in this aging population, our findings aim to inform targeted vision preservation strategies while contributing to global debates on myopia definitions in epidemiological contexts.

## Materials and Methods

**Ethics Statement**

The Fujian Eye Study (FJES) was prospectively registered in the Chinese Clinical Trial Registry (Registration ID: ChiCTR2100043349) prior to participant recruitment. Ethical oversight was provided by the Institutional Review Board of Xiamen Eye Center, Xiamen University (Approval Code: XMYKZX-KY-2018-001), with written informed consent secured from all enrollees following detailed explanation of study objectives and procedures. Protocol implementation strictly adhered to the ethical principles for medical research involving human subjects established by the Declaration of Helsinki (2013 revision).

**Study design**

The Fujian Eye Study (FJES) employed a multistage random cluster sampling design. Clusters were defined as administrative villages/urban communities, stratified by coastal-inland geography and urbanization level (urban/rural) based on 2017 census data.^[18]^ Of 120 selected clusters, 10,044 eligible residents were invited, with 8,211 participating (response rate: 81.7%). Non-participants did not differ significantly in age or sex distribution (p=0.12).

**On-site examination**

The report encompasses the following key components: a questionnaire addressing factors such as age, sex, level of urbanization, geographic location, educational background, income, phone usage habits (including usage in low-light environments), history of chronic diseases, and consumption of alcohol, tobacco, and tea. Additionally, it includes measurements of height, weight, body mass index (BMI), systolic blood pressure (SBP), diastolic blood pressure (DBP), heart rate (HR), intraocular pressure (IOP), presenting distance visual acuity (PDVA), best-corrected visual acuity (BCVA), and refractive status.

An experienced technician measured participants’ IOP with the handheld iCare rebound tonometer (RBT; Icare TA 01, Finland). Quintic measurements were obtained, and their mean was recorded and taken for further statistical analysis.

**Refractive Assessment Protocol**

Objective refraction parameters—including spherical power (DS), cylindrical power (DC), and axis orientation—were quantified using the Topcon KR800 autorefractor (Topcon Corporation, Tokyo, Japan). Three consecutive measurements per eye were obtained with the arithmetic mean adopted as the definitive refractive value to minimize intraoperator variability.

Visual function evaluation incorporated:

PDVA: Monocular assessment at 5 meters using the E-chart;

BCVA: Retesting with optimal spherocylindrical correction determined through trial frame refinement.

Operational Definition of High Myopia

Subjects were classified with high myopia when exhibiting spherical equivalent (SE) ≤−6.00 diopters,^[19]^ calculated as:

SE=Spherical power+Cylindrical power/2

**Statistical Methodology**

Data processing and analyses were executed using Stata/SE v15.1 (StataCorp LLC, TX, USA). Categorical variables were initially assessed through Pearson's χ² test with Yates' continuity correction where appropriate.

Univariate logistic regression models were first constructed to screen potential risk factors related with high myopia, with effect sizes expressed as odds ratios (ORs) and 95% confidence intervals (CIs). Multivariable analysis subsequently employed generalized linear mixed models incorporating cluster-robust standard errors to account for intra-cluster correlations inherent in the sampling design. Statistical significance was defined a priori at two-tailed p<0.05, with all estimates reported to three decimal places to enhance precision.

## Results

**Characteristics of the participants**

A total of 8,211 residents aged 50 and older were surveyed, and after excluding those with incomplete data, 8,024 individuals were included in the study. Among the participants, 309 (3.85% [95% CI: 3.43%-4.27%]) were identified as having high myopia. Of these, 179 (57.9%) were female, 177 (57.3%) resided in urban areas, and 222 (71.8%) lived in coastal regions. The prevalence of high myopia across the primary subgroups is summarized in Table 1.

| Table 1 The characteristics of the study participants and the prevalence of high myopia (n=8024) | | | | | |
| --- | --- | --- | --- | --- | --- |
| Characteristics | | Total population | High myopia | Prevalence (%) | p-value |
| Age (years) group | 50 to 54 | 1139 | 89 | 7.25 | <0.001 |
|  | 55 to 59 | 1323 | 41 | 3.01 |  |
|  | 60 to 64 | 1529 | 41 | 2.61 |  |
|  | 65 to 69 | 1583 | 59 | 3.59 |  |
|  | 70 to 74 | 1126 | 41 | 3.51 |  |
|  | 75 to 79 | 560 | 26 | 4.44 |  |
|  | 80 and above | 455 | 12 | 2.57 |  |
|  | Total | 8024 | 309 | 3.85 |  |
| Sex | Male | 3273 | 130 | 3.97 | 0.640 |
|  | Female | 4751 | 179 | 3.77 |  |
|  | Total | 8024 | 309 | 3.85 |  |
| Urbanization | Urban | 4583 | 177 | 3.86 | 0.952 |
|  | Rural | 3441 | 132 | 3.84 |  |
|  | Total | 8024 | 309 | 3.85 |  |
| Geographic location | Coastal | 6303 | 222 | 3.52 | 0.003 |
|  | Inland | 1721 | 87 | 5.06 |  |
|  | Total | 8024 | 309 | 3.85 |  |
| Education level | Illiteracy | 1242 | 34 | 2.74 | <0.001 |
|  | Primary school | 1487 | 45 | 3.03 |  |
|  | Middle school | 3039 | 92 | 3.03 |  |
|  | College and above | 1178 | 102 | 8.66 |  |
|  | Total | 6946 | 273 | 3.93 |  |
| Income level | <=2000 | 2352 | 69 | 2.93 | <0.001 |
|  | 2000-5000 | 1918 | 83 | 4.33 |  |
|  | >5000 | 579 | 41 | 7.08 |  |
|  | Total | 4849 | 193 | 3.98 |  |
| Duration of phone use (hours, h) | 0 | 2136 | 70 | 3.28 | 0.003 |
|  | < 1 | 2631 | 93 | 3.53 |  |
|  | 1 to 2 | 1030 | 45 | 4.37 |  |
|  | >2 | 1005 | 59 | 5.87 |  |
|  | Total | 6802 | 267 | 3.93 |  |

**Correlations Between High Myopia and Sociodemographic Characteristics**

In the overall study population, high myopia (OR = 0.686, P = 0.004) showed a significant association with geographic location, with a notably lower prevalence observed in coastal areas compared to inland regions. Conversely, high myopia (OR = 1.01, P = 0.952) demonstrated no significant correlation with urbanization, whether comparing urban areas to rural ones.

High myopia was found to be strongly associated with higher education levels (OR = 1.549, P < 0.001) and higher income (OR = 1.576, P < 0.001). As education and income levels increased, the prevalence of high myopia correspondingly rose. Furthermore, individuals who frequently used their phones exhibited a higher prevalence of high myopia (OR = 1.229, P < 0.001), with the rate increasing in relation to prolonged phone usage. The prevalence was notably higher among those who used their phones in dark environments (OR = 1.415, P = 0.008) compared to those who did not.

**Correlations Between High Myopia and Biological Characteristics**

In general, high myopia was strongly associated with younger age (OR = 1.024, P < 0.001), with its highest prevalence occurring among individuals aged 50–54 years. After the age of 55, the prevalence of high myopia appeared to stabilize. No significant associations were found between high myopia and sex (OR = 0.947, P = 0.640), tobacco use (OR = 1.232, P = 0.106), alcohol consumption (OR = 0.822, P = 0.188), or tea consumption (OR = 1.104, P = 0.363).

Height showed a significant association with high myopia (OR = 1.018, P = 0.015), with its prevalence rising alongside an increase in height. However, no significant correlation was observed with weight (OR = 1.003, P = 0.606) or BMI (OR = 0.9846, P = 0.382).

High myopia showed no correlation with SBP (OR = 1.000, P = 0.985), DBP (OR = 1.001, P = 0.756), or HR (OR = 1.009, P = 0.069). Additionally, it was found to be independent of any history of chronic diseases, including hypertension (OR = 0.963, P = 0.745), diabetes (OR = 0.897, P = 0.408), and hyperlipidemia (OR = 1.118, P = 0.481).

A strong correlation was identified between high myopia and elevated IOP (OR = 1.085, P < 0.001), with the prevalence of high myopia rising in tandem with increasing IOP levels.

**Multiple logistic regression**

A multiple logistic regression analysis was used to evaluate various parameters, including age, geographic location (coastal vs. inland), height, intraocular pressure (IOP), education level, income, phone usage duration, and phone use in darkness. The findings, summarized in Table 2, revealed that, after adjustments, high myopia was significantly associated with inland residency, elevated IOP and higher education levels. In contrast, age, height, income, phone usage duration, and phone use in darkness no longer emerged as significant predictors.

| Table 2 The multiple logistic regression in high myopia with several research factors in Fujian Eye Study | | | |
| --- | --- | --- | --- |
| Research factors | High myopia | | |
|  | Odds Ratio | 95% Confidential Interval (CI) | P-value |
| Geographic location | 0.690 | 0.47 to 1.00 | 0.049 |
| Age | 0.98 | 0.96 to 1.00 | 0.071 |
| Height | 0.98 | 0.96 to 1.01 | 0.138 |
| IOP | 1.07 | 1.03 to 1.11 | 0.001 |
| Education | 1.71 | 1.34 to 2.19 | <0.001 |
| Income | 1.06 | 0.81 to 1.39 | 0.674 |
| Phone use in the dark | 0.94 | 0.66 to 1.35 | 0.750 |
| Phone use or not | 0.60 | 0.34 to 1.05 | 0.075 |
| Duration of phone use | 1.24 | 1.00 to 1.53 | 0.052 |

Discussion

It is projected that by 2050, 9.8% of the population worldwide will have high myopia.^[8,20]^ The development of high myopia is largely influenced by a combination of genetic, environmental, behavioral, and other contributing factors. This cross-sectional eye study examined the prevalence of high myopia and its related factors, including biological and sociodemographic variables, among adults aged ≧ 50 years in Southern China. The analysis explored a broad range of potential contributing elements, such as age, sex, urbanization, geographic location, education level, income, height, weight, SBP, DBP, IOP, and medical histories of diabetes, hypertension, or hyperlipidemia. Additionally, lifestyle habits like phone use duration, phone use in the dark, and the consumption of tobacco, alcohol, or tea were also investigated. In this study, we identified significant correlations between high myopia and living in inland areas, lower educational attainment, and elevated intraocular pressure.

**The Prevalence of High Myopia**

A systematic review and meta-analysis of 145 articles revealed that only 59 studies explicitly defined and measured high myopia. Among these, 30.5% defined high myopia as -6.00 D or less, another 30.5% as less than -6.00 D, 35.6% as -5.00 D or less, while 1.7% defined it as -8.00 D or less, and an additional 1.7% as -3.00 D or less.^[8]^ Williams et al. conducted a multinational epidemiological investigation through the European Eye Epidemiology (E3) Consortium, aggregating refractive error data from fifteen population-based studies spanning multiple European regions. Their analysis employing the 2010 European Standard Population (adjusted for subjects aged 25-89 years) revealed an age-adjusted prevalence rate of 2.7% (95% confidence interval 2.69-2.73) for high myopia within the adult European population.^[10]^
The International Myopia Institute (IMI) has recommended defining high myopia as a spherical equivalent refractive error of ≤ -6.00 D when ocular accommodation is relaxed.^[17]^ In alignment with IMI’s guidelines, our study also adopted the threshold of -6.00 D or less to classify high myopia. Consistency in research standards is essential for ensuring comparability in big data analyses and for yielding more robust and convincing findings in future studies.

The prevalence of high myopia varies widely due to various limiting factors, including differences in countries, regions, age ranges, gender, and definitions.^[4–16]^ Moreover, there are relatively few reports addressing high myopia among the elderly population.^[4-7,13–16]^ Our study found that the prevalence of high myopia among adults aged ≧ 50 years in Southeast China was 3.85%. One segment of the CNHS study involving Yi and Han adults aged from 40 to 80 years old in Yunnan reported a prevalence of high myopia at 2.64% (95% CI, 1.75–3.53%).^[13]^ In another part of the CNHS, conducted among Mongolian and Han adults aged from 40 to 80 years old in Inner Mongolia, the prevalence of high myopia (SE < −6.0D) was recorded at 3.6% (95% CI, 2.8–4.4%).^[21]^ Similarly, a cross-sectional study of Yugur and Han adults aged from 40 to 80 years old living in Gansu, Northwest China, reported a prevalence of high myopia (SE < −6.0D) at 3.6%.^[22]^ In eastern China, a community-based cross-sectional survey of residents aged ≧ 60 years in a rural setting revealed that the age-adjusted prevalence of high myopia was 2.5% (95% CI, 2.1–2.9).^[23]^ In comparison, the Tajimi Study ( residents aged ≧ 40 years) showed that the prevalence of high myopia (SE < −5.0 diopters) was noticeably higher at 8.2% (95% CI, 7.2–9.2).^[11]^

**Factors Linked to High Myopia**

Two decades ago, multiple reports indicated that urban residents, individuals with higher education levels, and computer users were more prone to myopia, with the condition also being associated with higher intelligence.^[24–29]^ A cross-sectional study comparing Yugur and Han populations found a lower prevalence of high myopia in Yugur participants compared to Han participants (0.7% vs 5.0%, p < 0.001). High myopia was negatively associated with the Yugur population, aging, and being born in rural areas, while positively associated with a family history of myopia,^[22]^ which also aligned with our findings. The CNHS in Han and Yi populations aged from 40 to 80 years old years in Yunnan revealed that the Yi population had a lower prevalence of high myopia compared to the Han population (1.31% vs 3.34%, p = 0.049). In this study, myopia was found to be associated with ethnicity, age, height, time spent in rural areas, higher education level, and diabetes.^[13]^ Similar results were observed in another CNHS analysis involving Han and Mongolian populations aged 40–80 in Inner Mongolia.^[21]^ Nonetheless, our study showed that high myopia was not associated with age, height, urbanization, or certain features of educational relevance. This may be attributed to multiple interacting mechanisms: In aging populations, nuclear sclerosis-induced hyperopic shift may counterbalance pre-existing myopic progression, particularly in early-onset high myopia. This biomechanical equilibrium could obscure age-dependent trends, as observed in the Beijing Eye Study.[34] While taller stature correlates with longer axial length in adolescents, this relationship attenuates in older adults due to age-related scleral remodeling and vitreous degeneration. Our findings align with the Singapore Longitudinal Aging Study showing null height-myopia associations post-60 years.[35] Severe high myopia patients with early-onset complications (e.g., retinal detachment) may have been excluded from this community-based sample due to mobility limitations or prior hospitalization, truncating the observable risk spectrum. All these interpretations underscore the necessity of lifespan myopia trajectory analyses and refined exposure metrics in aging populations.

The JPHC-NEXT Eye Study investigated adults (age ≧ 40 years) without ocular surgery history. High myopia was defined as SE ≤ −6.00 diopters, based on the criteria set by AAO. The study found that the prevalence of high myopia was 3.8% in males and 5.9% in females, revealing a significant difference.^[30]^ However, our study reported no significant sex-related differences. While age was inversely associated and IOP positively associated, no correlations were observed between high myopia and other factors, including corneal structure, corneal endothelial cell density, height, BMI, HR, BP, biochemical profile, or current systemic and ocular disorders in either sex.^[30]^ Furthermore, our findings indicated the association between high myopia and IOP, but no association with age. This may be related to axial myopia and scleral thinning, or measurement errors such as the influence of corneal thickness on IOP measurement.^[31-32]^

The cross-sectional NHNES study suggested that myopia is linked to higher education levels in the U.S.,^[29]^ aligning with our findings. In contrast, both the Beijing Eye Study and the Central India Eye and Medical Study indicated no significant difference or even lower educational levels among the highly myopic group compared to the nonhighly myopic group.^[33]^ The inconsistent associations between educational attainment and high myopia across studies may stem from multifaceted interactions of methodological, demographic, and biological factors, such as the regional difference, the racial difference, gene-environment interactions, and unequal access to vision care. All these findings highlight context-dependent "environmental sensitivity thresholds" shaping education-myopia relationships, remind us that future research needs to comprehensively focus on the impact of multiple factors.

**Limitations**

This investigation has several methodological constraints. First, the cross-sectional design inherently restricts causal inference between identified risk factors and high myopia, as temporal relationships cannot be established. Second, the absence of axial length measurements—a gold-standard biomarker for pathological myopia—limits our ability to differentiate physiological high myopia from sight-threatening pathological forms, potentially obscuring clinical risk stratification. Third, this study lies in its focus on the prevalence of high myopia as defined, along with an analysis of related factors such as social demographics and systemic diseases. However, it does not include a correlation analysis of eye diseases, which we intend to address as the focal point of our future research.

In summary, this study is the first to report the prevalence of high myopia among adults (age ≧ 50 years) in Fujian Province, China. The prevalence rate was found to be 3.85%, and it was associated with inland residency, higher levels of education, and elevated IOP. We recommend integrating axial length measurements in future surveys and prioritizing vision screening for inland populations with limited healthcare access. Notably, the prevalence and contributing factors of high myopia differ across other racial groups and regions of China, potentially reflecting variations in environmental conditions, lifestyles, and genetic influences related to refractive errors. This research provides valuable epidemiological data to support regional policy-making efforts. To better understand the detailed associations between geographic location, IOP, educational background, and high myopia, further studies are needed across diverse age groups and ethnic populations, with a particular emphasis on investigating the underlying mechanisms.

## Declarations

## Ethics approval and consent to participate

## A clinical study registry was obtained for the 2018–2019 FJES study (Clinical trial number: ChiCTR2100043349, registration date: 2021-02-21) and the study protocol was approved by the Ethics Committee of Xiamen Eye Center afliated with Xiamen University (Acceptance No. XMYKZX-KY-2018-001).

## Data availability statement

## Data cannot be shared publicly because of incomplete use of the original data. Data are available from the corresponding author (contact via drlixiaoxin@163.com) for researchers who meet the criteria for access to confidential data.

## Conflict of interest statement

## All authors have participated in (a) conception and design, or analysis and interpretation of the data; (b) drafting the article or revising it critically for important intellectual content; and (c) approval of the final version. This manuscript has not been submitted to, nor is under review at, another journal or other publishing venue. All authors declare that they have no competing interests.

## Funding

## This study was supported by the National Natural Science Foundation of China (NSFC, No.81870672) and the Xiamen Health High Quality Development Science and Technology Plan for Youth and Middle aged Backbone Talents Training Project (No.2024GZL-GG83). The funding organization had no role in the study design, collection, analysis and interpretation of data.

## Author’s Contributions

## Yang Li took part in all parts of the study, including the study design and implementation, data collection and analysis, article writing and revision. Qinrui Hu completed the project together and participated in the revision of this article. Bin Wang mainly assisted in article revision and plotting. Xiangdong Luo and Mingqin Zhang were mainly responsible for patient management. Xiaoxin Li provided this project and oversaw the whole research.

## Acknowledgments

## We thank all FJES group members (Zhenglingling Yao, Liting Wang, Yi Liu, Wufu Qiu, Menging Lin, Yanhong Zhang) who made tremendous efforts to make the study successful, especially in the field examinations and data collection.

## Author details

^1^Eye Institute and Affiliated Xiamen Eye Center of Xiamen University, School of Medicine, Xiamen University, Xiamen, China.

^2^Fujian Provincial Key Laboratory of Corneal & Ocular Surface Diseases, Xiamen, Fujian, China.

^3^Xiamen Municipal Key Laboratory of Corneal & Ocular Surface Diseases, Xiamen, Fujian, China.

^4^Xiamen Research Center for Eye Diseases and Key Laboratory of Ophthalmology, Xiamen, Fujian, China.

^5^Department of Ophthalmology, Peking University People’s Hospital, Beijing100044, China.

## REFERENCES

[1] Flaxman SR, Bourne RRA, Resnikoff S, Ackland P, Braithwaite T, Cicinelli MV, Das A, Jonas JB, Keeffe J, Kempen JH, Leasher J, Limburg H, Naidoo K, Pesudovs K, Silvester A, Stevens GA, Tahhan N, Wong TY, Taylor HR; Vision Loss Expert Group of the Global Burden of Disease Study. Global causes of blindness and distance vision impairment 1990-2020: a systematic review and meta-analysis. Lancet Glob Health. 2017 Dec;5(12):e1221-e1234. doi: 10.1016/S2214-109X(17)30393-5. Epub 2017 Oct 11. PMID: 29032195.
[2] Ikuno, Y.; Ohji, M. High Myopia and the Vitreoretina; Complocations. In Retina (Philadelphia, PA), 5th ed.; Ryan, S.J., Ed.; Elsevier: Los Angeles, CA, USA, 2013; Volume 3, pp. 1912–1919.
[3] Wong, T. Y. et al. Epidemiology and disease burden of pathologic myopia and myopic choroidal neovascularization: an evidencebased systematic review. Am J Ophthalmol. 157, 9–25 e12 (2014).
[4] Evans JR, Fletcher AE, Wormald RP, et al. Causes of visual impairment in people aged 75 years and older in Britain: an add-on study to the MRC Trial of assessment and management of older people in the community. Br J Ophthalmol. 2004;88:365e370.
[5] Kelliher C, Kenny D, O’Brien C. Trends in blind registration in the adult population of the Republic of Ireland 1996e2003. Br J Ophthalmol. 2006;90:367e371.
[6] Zhao J, Xu X, Ellwein LB, et al. Causes of visual impairment and blindness in the 2006 and 2014 nine-province surveys in rural China. Am J Ophthalmol. 2019;197:80e87.
[7] Xu L, Li J, Cui T, et al. Refractive error in urban and rural adult Chinese in Beijing. Ophthalmology. 2005;112(10):1676–1683.
[8] Holden BA, Fricke TR, Wilson DA, Jong M, Naidoo KS, Sankaridurg P, Wong TY, Naduvilath TJ, Resnikoff S. Global Prevalence of Myopia and High Myopia and Temporal Trends from 2000 through 2050. Ophthalmology. 2016 May;123(5):1036-42. doi: 10.1016/j.ophtha.2016.01.006. Epub 2016 Feb 11.
[9] Morgan IG, French AN, Ashby RS, Guo X, Ding X, He M, Rose KA. The epidemics of myopia: Aetiology and prevention. Prog Retin Eye Res. 2018 Jan;62:134-149. doi: 10.1016/j.preteyeres.2017.09.004. Epub 2017 Sep 23. PMID: 28951126.
[10] Williams KM, Verhoeven VJ, Cumberland P, Bertelsen G, Wolfram C, Buitendijk GH, Hofman A, van Duijn CM, Vingerling JR, Kuijpers RW, Höhn R, Mirshahi A, Khawaja AP, Luben RN, Erke MG, von Hanno T, Mahroo O, Hogg R, Gieger C, Cougnard-Grégoire A, Anastasopoulos E, Bron A, Dartigues JF, Korobelnik JF, Creuzot-Garcher C, Topouzis F, Delcourt C, Rahi J, Meitinger T, Fletcher A, Foster PJ, Pfeiffer N, Klaver CC, Hammond CJ. Prevalence of refractive error in Europe: the European Eye Epidemiology (E(3)) Consortium. Eur J Epidemiol. 2015 Apr;30(4):305-15. doi: 10.1007/s10654-015-0010-0. Epub 2015 Mar 18. PMID: 25784363; PMCID: PMC4385146.
[11] Suzuki Y, Yamamoto T, Araie M, Iwase A, Tomidokoro A, Abe H, Shirato S, Kuwayama Y, Mishima HK, Shimizu H, Tomita G, Inoue Y, Kitazawa Y. [Tajimi Study review]. Nippon Ganka Gakkai Zasshi. 2008 Dec;112(12):1039-58. Japanese. PMID: 19157025.
[12] Rudnicka AR, Kapetanakis VV, Wathern AK, et al. Global variations and time trends in the prevalence of childhood myopia, a systematic review and quantitative meta-analysis: implications for aetiology and early prevention. Br J Ophthalmol. 2016;100(7):882-890.
[13] Wang M, Cui J, Shan G, Peng X, Pan L, Yan Z, Zhang J, Zhong Y, Ma J. Prevalence and risk factors of refractive error: a cross-sectional Study in Han and Yi adults in Yunnan, China. BMC Ophthalmol. 2019 Jan 25;19(1):33. doi: 10.1186/s12886-019-1042-0. PMID: 30683073; PMCID: PMC6347814.
[14] Ueda E, Yasuda M, Fujiwara K, et al. Trends in the preva- lence of myopia and myopic maculopathy in a Japanese population: the Hisayama study. Investig Ophthalmol Vis Sci. 2019;60(8):2781–2786.
[15] Williams KM, Bertelsen G, Cumberland P, et al. Increasing prevalence of myopia in Europe and the impact of education. Ophthalmology. 2015;122(7):1489–1497.
[16] Hashemi H, Khabazkhoob M, Jafarzadehpur E, Yekta AA, Emamian MH, Shariati M, Fotouhi A. High prevalence of myopia in an adult population, Shahroud, Iran. Optom Vis Sci. 2012 Jul;89(7):993-9. doi: 10.1097/OPX.0b013e31825e6554. PMID: 22705774.
[17] Flitcroft DI, He M, Jonas JB, Jong M, Naidoo K, Ohno-Matsui K, Rahi J, Resnikoff S, Vitale S, Yannuzzi L. IMI - Defining and Classifying Myopia: A Proposed Set of Standards for Clinical and Epidemiologic Studies. Invest Ophthalmol Vis Sci. 2019 Feb 28;60(3):M20-M30. doi: 10.1167/iovs.18-25957. PMID: 30817826; PMCID: PMC6735818.
[18] National Bureau of Statistics People’s Republic of China. Major Figures of the 2010 population census. Available at: http://www.stats.gov.cn/tjsj/pcsj/rkpc/6rp/indexch.htm. Accessed 03 Mar 2021.
[19] 中华医学会眼科学分会眼视光学组,中国医师协会眼科医师分会眼视光专业委员会,中国非公立医疗机构协会眼科专业委员会视光学组等. 高度近视防控专家共识（2023）[J]. 中华眼视光学与视觉科学杂志, 2023,25(6): 401-407.
[20] Baird PN, Saw SM, Lanca C, Guggenheim JA, Smith Iii EL, Zhou X, Matsui KO, Wu PC, Sankaridurg P, Chia A, Rosman M, Lamoureux EL, Man R, He M. Myopia. Nat Rev Dis Primers. 2020 Dec 17;6(1):99. doi: 10.1038/s41572-020-00231-4. PMID: 33328468.
[21] Wang M, Ma J, Pan L, Chen T, Wang HL, Wang YH, Wang WR, Pan XD, Qian YG, Zhang X, Zhong Y, Shan GL. Prevalence of and risk factors for refractive error: a cross-sectional study in Han and Mongolian adults aged 40-80 years in Inner Mongolia, China. Eye (Lond). 2019 Nov;33(11):1722-1732. doi: 10.1038/s41433-019-0469-0. Epub 2019 Jun 3. PMID: 31160702; PMCID: PMC7002754.
[22] Wang X, He H, Wang X, Shan G, Tao Z, Pan L, Li J, Ren X, Zhao H, Pan Z, Wang M, Zhong Y, Ma J. Prevalence and risk factors of myopia in Han and Yugur older adults in Gansu, China: a cross-sectional study. Sci Rep. 2020 May 19;10(1):8249. doi: 10.1038/s41598-020-65078-x. PMID: 32427926; PMCID: PMC7237487.
[23] Xu C, Pan C, Zhao C, Bi M, Ma Q, Cheng J, Song E. Prevalence and risk factors for myopia in older adult east Chinese population. BMC Ophthalmol. 2017 Oct 13;17(1):191. doi: 10.1186/s12886-017-0574-4. PMID: 29029601; PMCID: PMC5640960.
[24] Williams C, Miller LL, Gazzard G, et al. A comparison of measures of reading and intelligence as risk factors for the development of myopia in a UK cohort of children. Br J Ophthalmol 2008;92:1117–1121.
[25] Ip JM, Rose KA, Morgan IG, et al. Myopia and the urban environment: Findings in a sample of 12-year-old Australian school children. Invest Ophthalmol Vis Sci 2008;49:3858–3863.
[26] Williams C, Miller LL, Gazzard G, et al. A comparison of measures of reading and intelligence as risk factors for the development of myopia in a UK cohort of children. Br J Ophthalmol 2008;92:1117–1121.
[27] Lee YY, Lo CT, Sheu SJ, et al. What factors are associated with myopia in young Adults? A survey study in Taiwan military conscripts. Invest Ophthalmol Vis Sci 2013;54:1026–1033.
[28] Cortinez MF, Chiappe JP, Iribarren R. Prevalence of refractive errors in a population of offifice-workers in Buenos Aires, Argentina. Ophthalmic Epidemiol 2008;15:10–16.
[29] Nickels S, Hopf S, Pfeiffer N, Schuster AK. Myopia is associated with education: Results from NHANES 1999-2008. PLoS One. 2019 Jan 29;14(1):e0211196. doi: 10.1371/journal.pone.0211196. PMID: 30695049; PMCID: PMC6350963.
[30] Mori K, Kurihara T, Uchino M, Torii H, Kawashima M, Sasaki M, Ozawa Y, Yamagishi K, Iso H, Sawada N, Tsugane S, Yuki K, Tsubota K. High Myopia and Its Associated Factors in JPHC-NEXT Eye Study: A Cross-Sectional Observational Study. J Clin Med. 2019 Oct 25;8(11):1788. doi: 10.3390/jcm8111788. PMID: 31731571; PMCID: PMC6912595.
[31] Du Y, Meng J, He W, Qi J, Lu Y, Zhu X. Complications of high myopia: An update from clinical manifestations to underlying mechanisms. Adv Ophthalmol Pract Res. 2024 Jun 21;4(3):156-163. doi: 10.1016/j.aopr.2024.06.003. PMID: 39036706; PMCID: PMC11260019.
[32] Jonas JB, Spaide RF, Ostrin LA, Logan NS, Flitcroft I, Panda-Jonas S. IMI-Nonpathological Human Ocular Tissue Changes With Axial Myopia. Invest Ophthalmol Vis Sci. 2023 May 1;64(6):5. doi: 10.1167/iovs.64.6.5. PMID: 37126358; PMCID: PMC10153585.
[33] Jonas JB, Xu L, Wang YX, Bi HS, Wu JF, Jiang WJ, Nangia V, Sinha A, Zhu D, Tao Y, Guo Y, You QS, Wu LJ, Tao LX, Guo XH, Ohno-Matsui K, Panda-Jonas S. Education-Related Parameters in High Myopia: Adults versus School Children. PLoS One. 2016 May 6;11(5):e0154554. doi: 10.1371/journal.pone.0154554. PMID: 27152764; PMCID: PMC4859491.
[34] Xu L, Zhang L, Xia CR, Li JJ, Hu LN, Ma K, Chen YJ, Fan GZ, Song WX, Shi YY, Zhang SY, Sun BC. [The prevalence and its effective factors of primary angle-closure glaucoma in defined populations of rural and urban in Beijing]. Zhonghua Yan Ke Za Zhi. 2005 Jan;41(1):8-14. Chinese. PMID: 15774106.
[35] Tan CS, Chan YH, Wong TY, Gazzard G, Niti M, Ng TP, Saw SM. Prevalence and risk factors for refractive errors and ocular biometry parameters in an elderly Asian population: the Singapore Longitudinal Aging Study (SLAS). Eye (Lond). 2011 Oct;25(10):1294-301. doi: 10.1038/eye.2011.144. Epub 2011 Jul 1. PMID: 21720418; PMCID: PMC3194304.
