# Supplementary material for: Prevalence and related factors of high myopia among adults aged 50 years and older in Fujian Eye Study
Source: PLoS One. 2026 Aug 3;21(8):e0355042. doi: 10.1371/journal.pone.0355042 (PMC13432112; doi:10.1371/journal.pone.0355042)
Supplement: Attachment — Submitted filename: 2026.01.19 Response to Reviewers.docx [file pone.0355042.s007.docx]

Dear Reviewers,

Thank you for your thorough and insightful review of our manuscript, "Prevalence and related factors of high myopia among adults aged 50 years and older in Fujian Eye Study", Submission ID: PONE-D-25-14251. We sincerely appreciate the time and effort you have dedicated to evaluating our work and providing constructive feedback. Your comments have been invaluable in helping us improve the clarity, rigor, and overall quality of the manuscript. We have carefully considered each of your points and have made corresponding revisions to the manuscript. Our point-by-point responses are detailed below.

**Reviewer 1**

Minor Issues:

Abstract:

1.Comment: Authors mention “blood type”. However, per the general understanding of blood type (as blood group), there are no records of data collection on blood type in the methods section, and no records of it in the results section as well. Can authors clarify what they mean by blood type and exclude it from the abstract to avoid confusion.
**Response:** Thank you for catching this inconsistency. The inclusion of "blood type" in the abstract was an oversight from an earlier draft. This variable was collected in the initial questionnaire but was not included in the analysis presented in this manuscript. We have removed "blood type" and "medication use" from the Methods sentence in the Abstract. The revised sentence now reads: "Participants completed a questionnaire covering education, income, medical history, lifestyle habits (including smoking, drinking, and tea consumption)."

Introduction:
2. Comment: On page 3, paragraph 2, references 10 and 11 cited do not appear to be supportive of claim stated as authors suggest. Reference 10 talks about myopia in Europe, whereas reference 11 is on primary open-angle glaucoma with no data on myopia. Authors should re-check their references.
**Response:** We sincerely apologize for these citation errors. We have carefully re-checked and corrected the references.
* The claim "80-90% among young adults versus 3.6-8.2% in those over 40" is supported by references 9 (Morgan et al., 2018) and a combination of data from the corrected reference for the Tajimi Study (now cited as Ref 10, Sawada et al., 2008, which reports high myopia prevalence) and other regional studies. We have revised the reference citations Ref 11 accordingly.
* The incorrect glaucoma reference has been replaced with the appropriate citation (Sawada et al., 2008, the Tajimi Study report in Japanese) which provides the high myopia prevalence data for Japan. The reference list has been updated and renumbered consecutively.

Materials and Methods:
3. Comment: Page 5, subsection “On-site examination”: What was the repeatability of the technician's measurement of IOP? How was intra-operator variability assessed?
**Response:** The iCare rebound tonometer (RBT) is designed to be easy to use and exhibits good operator internal repeatability in clinical settings. The device automatically forces five consecutive measurements and obtains the average value as the final intraocular pressure value. And the personnel who measure intraocular pressure are the same operator, which reduces operational errors. We have added clarification in the revised manuscript: “The iCare tonometer has demonstrated good operator internal repeatability in field studies. Perform by the same operator to improve reliability.”

4. Comment: Page 5, subsection “On-site examination”: How was the accuracy of the tonometer determined and how accurate was it? How repeatable were its readings?
   **Response:** The iCare TA01 tonometer used in our study has been validated with the Goldmann applanation tonometer (GAT) in multiple studies. Although there is some controversy in current reports, it cannot be denied that it still has its advantages in epidemiological investigation research and clinical practice. We added a sentence citing relevant validation literature: 'In randomized controlled and observational studies, the iCare rebound tonometer has been validated with the Goldmann flattening tonometer, showing good correlation and reproducibility.^[19-21]^'.
5. Comment: Page 5, subsection “On-site examination”: Were outliers included/excluded in the calculation of mean IOP measurements or were they replaced with additional measurements?
   **Response:** The iCare device's built-in software performs an initial quality check. Measurements with poor signal quality (indicated by the device) were automatically discarded and not included in the set of five valid readings required for the average. Therefore, outliers in the form of technically flawed readings were excluded at the point of acquisition. No post-hoc statistical outlier removal was performed on the final mean IOP values used for analysis. We added a sentence “Measurements with poor signal quality (indicated by the device) were automatically discarded and not included in the set of five valid readings required for the average.” in the revised manuscript.
6. Comment: Page 5, subsection “Refractive error assessment protocol”: How was the accuracy of the autorefractor determined and how accurate was it? How repeatable were its readings?
   **Response:** The Topcon KR800 autorefractor is a widely used, clinically validated instrument.Compared with retinoscopy, measurements obtained before and after cycloplegia with both ARs showed excellent reliability for evaluating spherical equivalent and Jackson cross-cylinder values. We have added this information: "The Topcon KR800 autorefractor is a validated instrument with high accuracy and repeatability for population-based studies."
7. Comment: Page 5, subsection “Refractive error assessment protocol”: Were outliers included/excluded in the calculation of the arithmetic mean refractive error or were they replaced with additional measurements?
   **Response:** Similar to the IOP protocol, the three consecutive autorefractor measurements were taken under controlled conditions. If a measurement was deemed unstable or an error message appeared, the technician would retake it. The arithmetic mean was calculated from three consecutive valid measurements. No statistical outlier removal was applied afterward to these subject-level means. And we added the sentence "If a measurement was deemed unstable or an error message appeared, the technician would retake it. The arithmetic mean was calculated from three consecutive valid measurements." in the revised manuscript.
8. Comment: Page 5, subsection “Refractive error assessment protocol”: Who and how many people did the trial frame refinement? If many, how was the inter-operator variability tested and what was it?
   **Response:** Trial frame refinement was performed by a trained and certified optometrists. To ensure consistency, the optometrist underwent standardized training using the same protocol before the study commenced. The protocol emphasized achieving the best-corrected visual acuity (BCVA) with the minimal minus sphere (or maximal plus sphere) principle. While a formal inter-operator variability assessment (e.g., Kappa statistic) was not conducted across all pairs, periodic checks and calibration sessions were held to maintain standardization. We have clarified this in the text: "BCVA was retested with optimal spherocylindrical correction determined through trial frame refinement by a trained optometrists who followed a standardized protocol."

Results:
9.  Comment: On page 7-8, under the subsections “Correlations Between High Myopia and Sociodemographic Characteristics” and “Correlations Between High Myopia and Biological Characteristics”, I recommend that authors present the full tabulated results for the correlation analyses (univariate logistic regression analyses).
**Response:** This is an excellent suggestion that enhances transparency. We have created a new Supplementary Table 2 titled "The univariate logistic regression of high myopia among each subgroups (n=8024)" which presents the Odds Ratio (OR), 95% Confidence Interval (CI), and P-value for every variable tested (sociodemographic, biological, and lifestyle factors). We now reference this table in the relevant Results sections: "The results of univariate logistic regression analyses for all examined factors are presented in Table 2." in the revised manuscript and original Table 2 revised as Table 3. Through the summary of the table, we have identified some writing errors and have corrected them in the manuscript. Thank you for your suggestions, which have also enabled us to discover these errors.

Discussion:
10. Comment: Page 10, paragraph 1, last line: The authors' results section seem to suggest the opposite of the claim stated here. From Table 1 and 2, higher educational level is reported to be significantly correlated with high myopia. However, the phrase “lower educational attainment” is contradictory. Can the authors clarify this?
**Response:** Thank you for highlighting this critical error. This was a typographical mistake. The sentence should correctly state that high myopia was associated with higher educational attainment. We have corrected it: "In this study, we identified significant correlations between high myopia and living in inland areas, higher educational attainment, and elevated intraocular pressure."

11. Comment: Page 11, subsection “Factors Linked to High Myopia”, first sentence: Reference 24 and 26 are a duplication of the same article.
    **Response:** Thank you for your careful review. We have corrected this duplication. Reference 26 has been removed, and subsequent references have been renumbered accordingly throughout the text and in the reference list.
12. Comment: Page 11, subsection “Factors Linked to High Myopia”: What is the alignment between the Yugur and Han populations study and the authors' current study, considering that the authors did not assess family history of myopia, and did not find any association between high myopia and urbanization/rural-living? The only similarity between the two studies, as I can tell, is in relation to aging, which was later found not to be correlated with high myopia after adjustments in the multiple logistic regression analyses. I suggest that authors rephrase this to avoid confusion and propagating misleading information.
    **Response:** We agree and thank you for this suggestion. And we have deleted this paragraph to more accurately discuss the cited study in the context of our own findings.
13. Comment: Page 11, subsection “Factors Linked to High Myopia”: What do the authors mean by certain features of educational relevance?
    **Response:** Thank you for your careful review. This phrasing was indeed unclear. We intended to refer to factors often correlated with education in our context, such as income and prolonged near-work (proxied by phone use duration), which became non-significant in the multivariable model. We have rephrased for clarity: "Nonetheless, our study showed that high myopia was independently associated with inland residency and higher education, but not with age, height, urbanization, income, or prolonged phone use."
14. Comment: Page 12, subsection “Factors Linked to High Myopia”: The statement “Severe high myopia patients with early-onset complications (e.g., retinal detachment) may have been excluded from this community-based sample due to mobility limitations or prior hospitalization, truncating the observable risk spectrum” appears to be a limitation of the study, and therefore, is misplaced, disrupting the flow of the manuscript. Authors should consider restructuring to enhance clarity and flow.
    **Response:** Thank you very much for your insightful advice. This point is indeed a study limitation related to potential selection bias. We have moved this sentence to the dedicated Limitations section as “Third, this study lies in its focus on the prevalence of high myopia as defined, along with an analysis of related factors such as social demographics and systemic diseases. However, it does not include a correlation analysis of eye diseases, for example, severe high myopia patients with early-onset complications (e.g., retinal detachment) may have been excluded from this community-based sample due to mobility limitations or prior hospitalization, truncating the observable risk spectrum, which we intend to address as the focal point of our future research.”.
15. Comment: Page 12, subsection “Factors Linked to High Myopia”: The sentence “The cross-sectional NHNES study suggested that myopia is linked to higher education levels in the U.S.,[29] aligning with our findings.” See comment 10.
    **Response:** Thank you for your reminding. With the correction from Comment #10 established (our finding is higher education), this sentence is now consistent and correct as written.
16. Comment: Page 12, subsection “Factors Linked to High Myopia”: References 30-35 need reformatting. References 34 and 35 appear to have been included later, and so it has been numbered wrongly. Authors should reformat references to ensure appropriate and orderly numbering.
    **Response:** Thank you for your reminding and we apologize for this error. The entire reference list has been carefully checked, duplicates removed (per comment 11), and all citations have been renumbered sequentially throughout the manuscript. The in-text citations and the reference list now correspond correctly.
17. Comment: Page 13, “All these findings highlight context-dependent "environmental sensitivity thresholds" shaping education-myopia relationships, remind us that future research needs to comprehensively focus on the impact of multiple factors”: This statement has grammatical issues. Authors should consider rephrasing to fix grammar and enhance clarity.
    **Response:** Thank you for your insightful advice. We have revised the sentence for better grammar and flow: "All these findings highlight the mutual influence of multiple factors on the correlation between education and myopia, reminding us that future research needs to comprehensively investigate multiple factors and more accurately reveal the correlation between diseases and factors."

18. Comment: ROC curves: Authors do not discuss figures 1 and 2 which are the ROC curves. I recommend that authors discuss what the curve means and their implications in terms of their findings.
**Response:** Thank you for this observation. You are correct that our initial draft included ROC curve analysis, which indicated that IOP (AUC = 0.5740, Fig. 1) and educational background (AUC = 0.6107, Fig. 2) showed modest predictive accuracy for high myopia. However, upon further methodological review, we recognized that educational level—being a categorical variable—is not ideally suited for ROC analysis, which is typically applied to continuous or ordinal predictors. Therefore, in the final version of the manuscript, we removed the ROC results to maintain methodological rigor.

If you consider it valuable to retain or briefly mention the ROC findings in the context of IOP (a continuous variable), we would be glad to add a succinct summary in the Results section, along with a note on the interpretational limits regarding education. Please let us know your preference, and we will revise accordingly.

**Fig 1.** The ROC curve and AUC between high myopia and intraocular pressure.

**Fig 2.** The ROC curve and AUC between high myopia and educational level.

Limitations:
19. Comment: Considering the time of the data collection, authors should address issues relating to the time-sensitivity of the results, and potential implication of outdated data.
Response: This is a valid point. We have added a paragraph to the Limitations section acknowledging this: "Fourth, the data for this study were collected between 2018 and 2019. While the prevalence of high myopia in older adults is unlikely to change dramatically within a 5-7 year period, secular trends in environmental factors (e.g., digital device use) among preceding generations could influence future estimates. Nevertheless, the identified associations between high myopia and factors like education and IOP are likely to remain relevant, and this study provides a crucial baseline for this aging cohort in Southern China."

Abstract

1.Methods:
A population-based cross-sectional eye study was conducted from May 2018 to October 2019, enrolling residents aged 50 and older. Participants completed a questionnaire covering education, income, blood type, medical history, medication use, lifestyle habits (including smoking, drinking, and tea consumption). They also underwent physical and ophthalmological examinations measuring height, weight, systolic blood pressure (SBP), diastolic blood pressure (DBP), heart rate (HR), refraction, intraocular pressure (IOP), and visual acuity (distance and best-corrected). Statistical analysis was performed using Stata software, and a multivariate logistic regression model was employed to identify factors associated with high myopia. The ROC curve was used to assess predictive accuracy.

Comment：What was the rationale for including blood type in the study? It is not clearly explained in the main text.

It would be advisable to remove survey questions that are not described in detail in the main text.

**Response:** Thank you for your reminding and advice. The inclusion of "blood type" in the abstract was an oversight from an earlier draft. This variable was collected in the initial questionnaire but was not included in the analysis presented in this manuscript. We have removed "blood type" and "medication use" from the Methods sentence in the Abstract. The revised sentence now reads: "*Participants completed a questionnaire covering education, income, medical history, lifestyle habits (including smoking, drinking, and tea consumption).*"

2.Results:
A total of 8,024 residents were included in the study. The overall prevalence of high myopia was 3.85% [95% CI: 3.43%-4.27%]. Multiple logistic regression showed that high myopia was significantly associated with higher IOP (OR=1.07, p=0.001) and higher education levels (OR=1.71, p<0.001).

Comment：Please elaborate on the potential direction of the association between high myopia and elevated IOP. It is important to discuss whether high IOP contributes to high myopia or vice versa, providing supporting references, in at least one dedicated paragraph in the Discussion section.

**Response:** Thank you for this important point regarding the direction of the association between high myopia and elevated intraocular pressure (IOP). We fully agree that clarifying the potential causal pathway is of significant interest. In the revised Discussion section, we have added a dedicated paragraph addressing this issue. We explicitly state that, “*As a cross-sectional study, our findings can demonstrate a statistical association but cannot establish causality or determine the direction of the relationship. Recent a review showed anatomically,thinner trabecular meshwork increases the risk of high IOP, conversely lamina cribrosa defects may offer a fluid outlet, potentially mitigating the pressure.^[38]^ While another review showed that scleral matrix remodeling has been shown to contribute to the biomechanical susceptibility of the sclera to accommodation-induced IOP fluctuations, resulting in reduced scleral thickness, axial length (AL) elongation, and axial myopia.^[39]^ Thus, longitudinal or interventional studies are needed to clarify any clarified causal relationship in the future.*” This limitation of the cross-sectional design is already acknowledged in the "Limitations" subsection of the Discussion.

3.Introduction

Myopia has emerged as a global public health challenge, with uncorrected refractive errors accounting for 53% of all vision impairment cases worldwide.^[1]^

Comment：Given the numerous recent multicenter studies on the epidemiological research of high myopia and its global concern, it is recommended to cite studies from within the past 5 years and rewrite this section accordingly.

**Response:** Thank you for your constructive suggestion. We agree that updating the introductory context with recent evidence is important to reflect the current epidemiological understanding of high myopia. Accordingly, we have revised the Introduction section by incorporating key multicentre studies and reviews from the past five years that highlight the growing global burden and public health significance of high myopia. The relevant references have been updated, strengthening the foundation and timeliness of our research rationale. We believe this revision better aligns the manuscript with contemporary literature.

4.The "myopia time bomb" phenomenon—where early-onset high myopia progresses to pathological forms in later life—demands urgent investigation into aging cohorts.^[12]^

Comment：The term "myopia time bomb" is not used in Reference 12 that you cited. Could you clarify its source? If it is a term coined by the authors themselves, please remove it to avoid misleading readers into thinking it is a newly coined term from Reference 12.

**Response:** Thank you for your attentive reading and for highlighting this discrepancy. You are correct that the term "myopia time bomb" was not directly used in Reference 12. It was an informal, summarizing phrase we used to conceptualize the described phenomenon of progression from early-onset to pathological myopia in later life. To avoid any potential confusion or misinterpretation for readers regarding its origin, we have removed this specific term from the manuscript as suggested. The sentence has been revised to describe the public health concern more directly, citing the appropriate literature for the underlying concept. The modified text is as follows:

“*The early-onset high myopia progresses to pathological forms in later life demands urgent investigation into aging cohorts.^[12]^*”

5.This operational definition underpins our study, which addresses three critical gaps:

1.Scarcity of data on high myopia among Chinese elderly;

2.Limited evidence regarding modifiable risk factors beyond genetic predisposition;

3.Regional healthcare disparities in Fujian Province, where coastal-inland socioeconomic gradients may influence outcomes.

By investigating prevalence patterns and associated factors in this aging population, our findings aim to inform targeted vision preservation strategies while contributing to global debates on myopia definitions in epidemiological contexts.

Comment：This section is helpful in clarifying the motivation of the study; however, it does not follow the manuscript’s formatting style. Converting the listed points (1), (2), and (3) into a single cohesive paragraph in narrative form would improve readability.

**Response:** Thank you for your suggestion regarding the formatting of this section. We agree that integrating the listed points into a cohesive narrative paragraph improves both readability and consistency with the manuscript's overall style. Accordingly, we have revised the text to present the three research gaps in a continuous, flowing paragraph format as recommended.

The modified text is as follows:

“*This operational definition underpins our study, which aims to address several critical gaps in the current literature. First, there remains a scarcity of population-based data on high myopia specifically among the elderly in China. Second, evidence regarding modifiable risk factors beyond genetic predisposition is still limited. Third, regional healthcare disparities within Fujian Province—where notable coastal-inland socioeconomic gradients exist—may influence ocular health outcomes, yet this has been underexplored. By investigating the prevalence patterns and associated factors in this aging cohort, our findings aim to inform targeted vision preservation strategies and contribute to the ongoing global discussion on defining and assessing myopia in epidemiological contexts.*”

## 6.Materials and Methods

**Ethics Statement**

The Fujian Eye Study (FJES) was prospectively registered in the Chinese Clinical Trial Registry (Registration ID: ChiCTR2100043349) prior to participant recruitment. Ethical oversight was provided by the Institutional Review Board of Xiamen Eye Center, Xiamen University (Approval Code: XMYKZX-KY-2018-001), with written informed consent secured from all enrollees following detailed explanation of study objectives and procedures. Protocol implementation strictly adhered to the ethical principles for medical research involving human subjects established by the Declaration of Helsinki (2013 revision).

Comment：This part is somewhat redundant and could be removed without affecting the overall message.

**Response:** Thank you for your suggestion. As recommended, we have removed the Ethics Statement paragraph to enhance the conciseness of the Methods section. We appreciate your guidance in streamlining the manuscript.

7.**Refractive Assessment Protocol**

Objective refraction parameters—including spherical power (DS), cylindrical power (DC), and axis orientation—were quantified using the Topcon KR800 autorefractor (Topcon Corporation, Tokyo, Japan). Three consecutive measurements per eye were obtained with the arithmetic mean adopted as the definitive refractive value to minimize intraoperator variability.

Visual function evaluation incorporated:

PDVA: Monocular assessment at 5 meters using the E-chart;

BCVA: Retesting with optimal spherocylindrical correction determined through trial frame refinement.

Operational Definition of High Myopia

Subjects were classified with high myopia when exhibiting spherical equivalent (SE) ≤−6.00 diopters,^[19]^ calculated as:

SE=Spherical power+Cylindrical power/2

Comment：it does not follow the manuscript’s formatting style. Converting the listed into a single cohesive paragraph in narrative form would improve readability.

**Response:** Thank you for your suggestion. We have revised the "Refractive Assessment Protocol" section to convert the previously listed items into a single, cohesive narrative paragraph, as recommended. All methodological details and the operational definition of high myopia, including the formula for spherical equivalent, have been preserved within the flowing text to improve readability and align with the manuscript's formatting style.

The modified text is as follows:

“*Objective refraction parameters—including spherical power (DS), cylindrical power (DC), and axis orientation—were quantified using the Topcon KR800 autorefractor (Topcon Corporation, Tokyo, Japan). The Topcon KR800 autorefractor is a validated instrument with high accuracy and repeatability for population-based studies.^[22]^ Three consecutive measurements per eye were obtained with the arithmetic mean adopted as the definitive refractive value to minimize intraoperator variability. If a measurement was deemed unstable or an error message appeared, the technician would retake it. The arithmetic mean was calculated from three consecutive valid measurements. Visual function evaluation incorporated monocular presentation distance visual acuity (PDVA), assessed at 5 meters using the E-chart, and best-corrected visual acuity (BCVA), determined by retesting with optimal spherocylindrical correction refined through a trial frame. Based on the calculated spherical equivalent (SE = spherical power + cylindrical power/2), participants were classified as having high myopia if SE was ≤ −6.00 diopters^[23]^.*”

| 8.Table 1 The characteristics of the study participants and the prevalence of high myopia (n=8024) | | | | | |
| --- | --- | --- | --- | --- | --- |
| Characteristics | | Total population | High myopia | Prevalence (%) | p-value |
| Age (years) group | 50 to 54 | 1139 | 89 | 7.25 | <0.001 |
|  | 55 to 59 | 1323 | 41 | 3.01 |  |
|  | 60 to 64 | 1529 | 41 | 2.61 |  |
|  | 65 to 69 | 1583 | 59 | 3.59 |  |
|  | 70 to 74 | 1126 | 41 | 3.51 |  |
|  | 75 to 79 | 560 | 26 | 4.44 |  |
|  | 80 and above | 455 | 12 | 2.57 |  |
|  | Total | 8024 | 309 | 3.85 |  |
| Sex | Male | 3273 | 130 | 3.97 | 0.640 |
|  | Female | 4751 | 179 | 3.77 |  |
|  | Total | 8024 | 309 | 3.85 |  |
| Urbanization | Urban | 4583 | 177 | 3.86 | 0.952 |
|  | Rural | 3441 | 132 | 3.84 |  |
|  | Total | 8024 | 309 | 3.85 |  |
| Geographic location | Coastal | 6303 | 222 | 3.52 | 0.003 |
|  | Inland | 1721 | 87 | 5.06 |  |
|  | Total | 8024 | 309 | 3.85 |  |
| Education level | Illiteracy | 1242 | 34 | 2.74 | <0.001 |
|  | Primary school | 1487 | 45 | 3.03 |  |
|  | Middle school | 3039 | 92 | 3.03 |  |
|  | College and above | 1178 | 102 | 8.66 |  |
|  | Total | 6946 | 273 | 3.93 |  |
| Income level | <=2000 | 2352 | 69 | 2.93 | <0.001 |
|  | 2000-5000 | 1918 | 83 | 4.33 |  |
|  | >5000 | 579 | 41 | 7.08 |  |
|  | Total | 4849 | 193 | 3.98 |  |
| Duration of phone use (hours, h) | 0 | 2136 | 70 | 3.28 | 0.003 |
|  | < 1 | 2631 | 93 | 3.53 |  |
|  | 1 to 2 | 1030 | 45 | 4.37 |  |
|  | >2 | 1005 | 59 | 5.87 |  |
|  | Total | 6802 | 267 | 3.93 |  |

Comment：Please consider adding horizontal lines between subgroups to clearly distinguish age variables from other factors such as sex and urbanization, which would improve the readability of the table；For consistency in number formatting, please revise “8024” to “8,024”.

The table should include a footnote specifying the statistical test or method used, so that readers can clearly understand how the results were obtained.

I would suggest grouping the data by decades (50s, 60s, 70s, and ≥80 years) rather than using 5-year age intervals. When stratified in 5-year increments, the prevalence does not show a clear overall trend and instead fluctuates, which makes it difficult to draw meaningful conclusions. Therefore, a more granular age stratification may not be necessary.

Could the authors clarify how “high school” was categorized or handled in the analysis?

Please clarify the unit of measurement for “income.” Is it monthly income? It would be helpful to express it in dollars to ensure clarity.

Given that average daily smartphone use among adolescents commonly exceeds 6 hours, and is generally more than 2 hours per day, the current categorization into 1- and 2-hour groups may not adequately reflect actual usage patterns. Reclassifying the groups and rerunning the analysis may provide more meaningful results.

For consistency in number formatting, please revise “8024” to “8,024”.

**Response:** Thank you for your detailed and constructive suggestions regarding Table 1. We have revised the table accordingly, and our specific responses to each point are as follows:

****Horizontal lines & number formatting:**** We have added horizontal lines between major variable subgroups (e.g., after Age, Sex, etc.) to improve visual distinction and readability. All instances of "8024" and similar numbers have been formatted with commas (e.g., 8,024) for consistency.

****Statistical test footnote:**** A footnote has been added to the table specifying: "P-values were derived from chi-square tests for trend (ordinal variables) or independence (nominal variables)."

****Age group stratification:**** We agree that grouping by decade provides a clearer presentation of trends for this aging cohort. The "Age (years) group" rows have been re-categorized as: **50–59**, **60–69**, **70–79**, and **≥80**. The corresponding data have been recalculated, and the p-value has been updated accordingly.

****Clarification on "high school" categorization:**** In our classification, both junior middle school and senior middle school (also known as high school) are collectively referred to as “middle school”.

****Clarification of income unit:**** The income levels refer to **monthly household income per capita in Chinese Yuan (CNY)**. We have added the unit of income in the income column.

****Categorization of phone use duration:**** We appreciate your careful review. While smartphone use patterns in adolescents are well-documented, our study focuses on an older population (age ≥50), whose usage patterns and reported durations differ significantly. Our categorization (0, <1, 1-2, >2 hours/day) was determined a priori based on the distribution of self-reported data in this specific study and aimed to capture meaningful variance within this age group. We acknowledge that alternative categorizations exist. To address your suggestion, we conducted a correlation analysis using the more detailed classification in the original questionnaire. The correlation is still significant (p=0.005), and the direction of action is consistent. Therefore, we chose to retain the current classification in the main table as it better reflects the dose-response patterns observed in our data. Should a more granular categorization be preferable, we can revise the table accordingly.

The revised Table 1 incorporating these changes is presented below. We believe these modifications have significantly enhanced the table's clarity, accuracy, and reader-friendliness.

9. In the overall study population, high myopia (OR = 0.686, P = 0.004) showed a significant association with geographic location, with a notably lower prevalence observed in coastal areas compared to inland regions. Conversely, high myopia (OR = 1.01, P = 0.952) demonstrated no significant correlation with urbanization, whether comparing urban areas to rural ones.

Comment：Please clarify to specify the statistical test or method used, so that readers can clearly understand how the results were obtained.

**Response:** Thank you for this important comment. In response to your suggestion to clarify the statistical methods used for these associations, we have revised the Results section accordingly. Specifically, we now explicitly state that univariate logistic regression was employed to assess the crude associations between each candidate variable (including geographic location and urbanization) and high myopia. To present these results more clearly and comprehensively, we have added a new Table 2 that summarizes the outcomes of all univariate logistic regression analyses. The textual description of the findings for geographic location and urbanization has been integrated with and now references this new table, ensuring readers can fully understand how these results were obtained.

The modified text is as follows:

“*The associations between various demographic, socioeconomic, lifestyle factors and high myopia were first assessed using univariate logistic regression. In the overall study population, high myopia (OR = 0.686, P = 0.004) showed a significant association with geographic location, with a notably lower prevalence observed in coastal areas compared to inland regions. Conversely, high myopia (OR = 1.007, P = 0.952) demonstrated no significant correlation with urbanization, whether comparing urban areas to rural ones.*”

10.Furthermore, individuals who frequently used their phones exhibited a higher prevalence of high myopia (OR = 1.229, P < 0.001), with the rate increasing in relation to prolonged phone usage.

Comment：Given that average daily smartphone use among adolescents commonly exceeds 6 hours, and is generally more than 2 hours per day, the current categorization into 1- and 2-hour groups may not adequately reflect actual usage patterns. Reclassifying the groups and rerunning the analysis may provide more meaningful results.

**Response:** Thank you for this thoughtful comment regarding the categorization of phone use duration. We appreciate the opportunity to clarify our methodological approach. In our original questionnaire, phone use duration was indeed collected in more granular categories: 0, <0.5, 0.5–1, 1–2, 2–4, 4–6, and >6 hours/day. However, as you correctly anticipated might be an issue in an older cohort, the distribution of participants across these categories was highly uneven, with very few respondents in the <0.5, 2–4, 4–6, and >6 hours/day groups. To ensure stable and reliable estimates in our statistical model, we consolidated these categories into the broader groups presented in the manuscript (0, <1, 1–2, >2 hours/day), which better reflected the meaningful distribution of usage within our specific study population (age ≥50 years).

To address your concern directly and test the robustness of our findings, we have rerun the analysis using the original, finer categories. The results confirmed a consistent, significant positive trend (P=0.003), with the point estimates aligning with our reported dose-response relationship. Therefore, while the statistical conclusion remains unchanged, we are happy to revise the table to present the data according to the original, more detailed classification if you believe it would provide more meaningful insight for readers, despite the small numbers in some categories. We would be grateful for your guidance on whether presenting the original categorization would be preferable in this context.

11.The prevalence was notably higher among those who used their phones in dark environments (OR = 1.415, P = 0.008) compared to those who did not.

Comment：Could the authors clarify where in the table the information on whether participants use smartphones in dark environments is presented?

**Response:** Thank you for your reminding, and the variable 'phone use in dark environments' in Table 2. We appreciate your careful review, which has helped improve the transparency and presentation of our results.

12.A strong correlation was identified between high myopia and elevated IOP (OR = 1.085, P < 0.001), with the prevalence of high myopia rising in tandem with increasing IOP levels.

Comment：This finding indicates a simple correlation between high myopia and elevated IOP, and does not imply any causal direction. The authors should discuss this point in more detail in the Discussion section, clarifying that it is not possible to determine which is the cause and which is the effect.

**Response:** Thank you for raising this critical point. We fully agree that the observed association between higher IOP and high myopia in our cross-sectional study indicates correlation and cannot establish causality or directionality. As you recommended, we have added a dedicated paragraph in the Discussion section to elaborate on this issue. The added text explicitly acknowledges the limitation of our study design in determining causality, reviews the existing literature discussing potential bidirectional mechanisms (e.g., IOP as a mechanical contributor to axial elongation vs. myopia-related ocular structural changes influencing IOP measurement), and concludes that longitudinal studies are required to clarify any causal relationship. We believe this addition provides the necessary context and caution in interpreting our findings regarding IOP.

13.Table 2 Phone use in the dark

Comment：The criteria for this variable are very ambiguous. It is difficult to define “dark” in terms of illumination level, duration, or specific environment. The authors should provide a more detailed explanation.

**Response:** Thank you for this important comment. We agree that the term "dark environments" requires a clear operational definition to ensure reproducibility and correct interpretation. We have revised the relevant section in the Methods to provide a more precise explanation.

“*The variable 'phone use in dark environments' was based on participant self-report in our questionnaire : 'Do you use mobile phone after turning off the lights at night?' Responses were recorded as a binary variable (Yes/No). This measure was intended to capture a common behavioral pattern rather than a precisely quantified photometric state.*”

We appreciate your feedback, which has helped us improve the clarity and rigor of our methodology.

14.Discussion

Williams et al. conducted a multinational epidemiological investigation through the European Eye Epidemiology (E3) Consortium, aggregating refractive error data from fifteen population-based studies spanning multiple European regions.

Comment：There is no need to abbreviate terms that are mentioned only once. Please review the manuscript and eliminate such unnecessary abbreviations.

**Response:** Thank you for your meticulous review of the discussion section and for pointing out the unnecessary use of abbreviations. We have carefully reviewed the entire manuscript and removed all non-essential abbreviations, including “E3 Consortium” in the mentioned sentence, ensuring that terms are written in full upon first use or when they appear infrequently. We appreciate this suggestion, which has enhanced the clarity and professionalism of the text.

15.The prevalence of high myopia varies widely due to various limiting factors, including differences in countries, regions, age ranges, gender, and definitions.^[4–16]^

Comment：Sex? Gender? unifying the terminology throughout the manuscript is necessary.

**Response:** Thank you for your careful attention to terminology. We sincerely apologize for the inconsistency in the use of "sex" and "gender" within the manuscript. We have now thoroughly reviewed the entire text and have standardized the terminology, using "sex" consistently throughout to refer to the biological category (male/female) of participants, in line with standard epidemiological reporting. We appreciate your vigilance in helping us improve the precision and consistency of our manuscript.

**16.Factors Linked to High Myopia**

Comment：The discussion is currently too detailed, obscuring the main message. The authors are encouraged to restructure it around key prior studies, provide in-depth explanation of the significant risk factors identified in their study, and emphasize their own interpretation, with careful attention to IOP.

**Response:** Thank you for this valuable suggestion regarding the structure and focus of the Discussion. We agree that the previous version was overly detailed, which may have obscured the core messages. In revising the section entitled "Factors Linked to High Myopia", we have undertaken a comprehensive restructuring. We now frame the discussion more concisely around the key and consistent findings from major prior studies, providing a clearer backdrop for our own results. The revised text then focuses on an in-depth explanation and interpretation of the significant risk factors identified in our study—particularly higher education levels, prolonged near-work/phone use, geographic location, and, with careful attention as you noted, the association with elevated IOP. We have streamlined the narrative to emphasize our own interpretation of these findings within the context of the existing literature and the specific characteristics of our aging cohort. We believe these changes have made the Discussion more focused, interpretive, and effective in highlighting the main contributions of our study.

17.中华医学会眼科学分会眼视光学组,中国医师协会眼科医师分会眼视光专业委员会,中国非公立医疗机构协会眼科专业委员会视光学组等. 高度近视防控专家共识（2023）[J]. 中华眼视光学与视觉科学杂志, 2023,25(6): 401-407.

Comment：Papers not written in English should be excluded from citations.

**Response:** Thank you for this correction. In accordance with your suggestion and standard academic practice, we have removed the cited non-English reference from the manuscript's reference list and substitute it with an appropriate English-language reference. We appreciate your guidance in ensuring the citations align with the journal's requirements.

We are truly grateful for your comprehensive review and insightful comments, which have been invaluable in refining our work. We believe that incorporating your suggestions has significantly enhanced the quality of the manuscript. We hope that our detailed responses and the revised version now meet with your approval. And we welcome any further guidance you may have.

Sincerely,

Yang Li

E-mail：[liyang686500@163.com](mailto:liyang686500@163.com)
