# Supplementary material for: Prevalence and related factors of high myopia among adults aged 50 years and older in Fujian Eye Study
Source: PLoS One. 2026 Aug 3;21(8):e0355042. doi: 10.1371/journal.pone.0355042 (PMC13432112; doi:10.1371/journal.pone.0355042)
Supplement: Attachment — Submitted filename: REVIEW 1 - PONE-D-25-14251_R1.docx [file pone.0355042.s006.docx]

**REVIEW - PONE-D-25-14251_R1**

The authors have addressed all the comments and concerns raised in the previous review. However, there are a few grammatical errors that impedes clarity and understanding of the manuscript. The authors are advised to employ the services of an experienced English writer to correct all errors and enhance the flow and clarity of the manuscript.

Again, the authors should cross-check every reference cited to make sure it is well numbered and correlated with the claims and statements made throughout the manuscript, since references have been updated.

Few specific instances of grammatical errors are cited below:

***Minor issues***

Abstract:

1. “Participants completed a questionnaire covering education, income, medical history, lifestyle habits (including smoking, drinking, and tea consumption).” Consider inserting an “and” between the last two items on the list: “……medical history, and lifestyle habits (including……”.

Materials and Methods:

2. “The iCare tonometer has demonstrated good operator internal repeatability in field studies. Perform by the same operator to improve reliability.” The second clause appears to stand alone which makes it incomplete.
3. "BCVA was retested with optimal spherocylindrical correction determined through trial frame refinement by a trained optometrists who followed a standardized protocol." Was it a single trained optometrist or multiple optometrists? It is not quite clear from the use of “a” and plural form “optometrists”.
