# Supplementary material for: Prevalence and related factors of high myopia among adults aged 50 years and older in Fujian Eye Study
Source: PLoS One. 2026 Aug 3;21(8):e0355042. doi: 10.1371/journal.pone.0355042 (PMC13432112; doi:10.1371/journal.pone.0355042)
Supplement: Peer review history

# Peer review: Decision Letter 0

**DOI:** 10.1371/journal.pone.0355042.r001

**Authors:** Hyojin Kim

6 Jan 2026

PONE-D-25-14251

Prevalence and related factors of high myopia among adults aged 50 years and older in Fujian Eye Study

PLOS One

Dear Dr. Li,

Thank you for submitting your manuscript to PLOS ONE. After careful consideration, we feel that it has merit but does not fully meet PLOS ONE’s publication criteria as it currently stands. Therefore, we invite you to submit a revised version of the manuscript that addresses the points raised during the review process.

Please submit your revised manuscript by Feb 20 2026 11:59PM. If you will need more time than this to complete your revisions, please reply to this message or contact the journal office at plosone@plos.org. When you're ready to submit your revision, log on to https://www.editorialmanager.com/pone/ and select the 'Submissions Needing Revision' folder to locate your manuscript file.

Please include the following items when submitting your revised manuscript:

- A letter that responds to each point raised by the academic editor and reviewer(s). You should upload this letter as a separate file labeled 'Response to Reviewers'.
- A marked-up copy of your manuscript that highlights changes made to the original version. You should upload this as a separate file labeled 'Revised Manuscript with Track Changes'.
- An unmarked version of your revised paper without tracked changes. You should upload this as a separate file labeled 'Manuscript'.

If you would like to make changes to your financial disclosure, please include your updated statement in your cover letter. Guidelines for resubmitting your figure files are available below the reviewer comments at the end of this letter.

If applicable, we recommend that you deposit your laboratory protocols in protocols.io to enhance the reproducibility of your results. Protocols.io assigns your protocol its own identifier (DOI) so that it can be cited independently in the future. For instructions see: https://journals.plos.org/plosone/s/submission-guidelines#loc-laboratory-protocols. Additionally, PLOS ONE offers an option for publishing peer-reviewed Lab Protocol articles, which describe protocols hosted on protocols.io. Read more information on sharing protocols at https://plos.org/protocols?utm_medium=editorial-email&utm_source=authorletters&utm_campaign=protocols.

We look forward to receiving your revised manuscript.

Kind regards,

Hyojin Kim

Academic Editor

PLOS One

**Journal Requirements:**

When submitting your revision, we need you to address these additional requirements.

1. Please ensure that your manuscript meets PLOS ONE's style requirements, including those for file naming. The PLOS ONE style templates can be found at

https://journals.plos.org/plosone/s/file?id=wjVg/PLOSOne_formatting_sample_main_body.pdf and

https://journals.plos.org/plosone/s/file?id=ba62/PLOSOne_formatting_sample_title_authors_affiliations.pdf

2. Thank you for stating the following financial disclosure:

“This study was supported by the National Natural Science Foundation of China (NSFC, No.81870672).”

Please state what role the funders took in the study.  If the funders had no role, please state: "The funders had no role in study design, data collection and analysis, decision to publish, or preparation of the manuscript."

If this statement is not correct you must amend it as needed.

Please include this amended Role of Funder statement in your cover letter; we will change the online submission form on your behalf.

3. Please note that funding information should not appear in any section or other areas of your manuscript. We will only publish funding information present in the Funding Statement section of the online submission form. Please remove any funding-related text from the manuscript.

4. In the online submission form you indicate that your data is not available for proprietary reasons and have provided a contact point for accessing this data. Please note that your current contact point is a co-author on this manuscript. According to our Data Policy, the contact point must not be an author on the manuscript and must be an institutional contact, ideally not an individual. Please revise your data statement to a non-author institutional point of contact, such as a data access or ethics committee, and send this to us via return email. Please also include contact information for the third party organization, and please include the full citation of where the data can be found.

5. Your ethics statement should only appear in the Methods section of your manuscript. If your ethics statement is written in any section besides the Methods, please delete it from any other section.

6. Please include a separate caption for each figure in your manuscript.

7. Please ensure that you refer to Figures 1, and 2 in your text as, if accepted, production will need this reference to link the reader to the figure.

8. Please upload a new copy of Figures 1 and 2 as the detail is not clear. Please follow the link for more information:  https://journals.plos.org/plosone/s/figures

9. If tables are embedded in the manuscript and ALSO loaded as separate files, please delete the separate files, leaving only the tables in the manuscript file.

10. If the reviewer comments include a recommendation to cite specific previously published works, please review and evaluate these publications to determine whether they are relevant and should be cited. There is no requirement to cite these works unless the editor has indicated otherwise.

**Additional Editor Comments:**

Thank you for submitting your manuscript to our journal. After peer review, we have determined that the manuscript requires major revision before it can be considered for publication.

Please revise the manuscript to address all reviewer comments and provide a detailed, point-by-point response outlining the changes made.

[Note: HTML markup is below. Please do not edit.]

Reviewers' comments:

Reviewer's Responses to Questions

**Comments to the Author**

1. Is the manuscript technically sound, and do the data support the conclusions?

The manuscript must describe a technically sound piece of scientific research with data that supports the conclusions. Experiments must have been conducted rigorously, with appropriate controls, replication, and sample sizes. The conclusions must be drawn appropriately based on the data presented.

Reviewer #1: Yes

Reviewer #2: Partly

**********

2. Has the statistical analysis been performed appropriately and rigorously?

Reviewer #1: Yes

Reviewer #2: Yes

**********

3. Have the authors made all data underlying the findings in their manuscript fully available?

The PLOS Data policy requires authors to make all data underlying the findings described in their manuscript fully available without restriction, with rare exception (please refer to the Data Availability Statement in the manuscript PDF file). The data should be provided as part of the manuscript or its supporting information, or deposited to a public repository. For example, in addition to summary statistics, the data points behind means, medians and variance measures should be available. If there are restrictions on publicly sharing data—e.g. participant privacy or use of data from a third party—those must be specified.

Reviewer #1: Yes

Reviewer #2: Yes

**********

4. Is the manuscript presented in an intelligible fashion and written in standard English?

PLOS ONE does not copyedit accepted manuscripts, so the language in submitted articles must be clear, correct, and unambiguous. Any typographical or grammatical errors should be corrected at revision, so please note any specific errors here.

Reviewer #1: Yes

Reviewer #2: Yes

**********

5. Review Comments to the Author

Please use the space provided to explain your answers to the questions above. You may also include additional comments for the author, including concerns about dual publication, research ethics, or publication ethics. (Please upload your review as an attachment if it exceeds 20,000 characters)

Reviewer #1: 1. Summary of the research

The study aimed to report the prevalence of high myopia in an older adult and elderly/aged population, as well as the factors associated with high myopia. This was to provide evidence that was lacking on high myopia among aged populations who are at higher risk of complications of high myopia. The authors report a prevalence of 3.85% in their population, with high myopia being significantly correlated with higher IOP and higher educational level. The authors conclude with a recommendation for increased eye care accessibility among the aged, especially those with higher IOP and educational attainment levels.

This research provides evidence on the prevalence and associated risk factors for high myopia in an aged population who are at higher risk for high myopia-associated ocular complications. This was previously lacking as previous literature mostly focused on younger populations.

A major strength of the study is related to the sampling: large sample size, high response rate (>80%) and report of non-significant variation between respondents and non-respondents of the study. However, a major limitation of this study is the time-sensitivity of the findings. The data was collected in 2018-2019, putting the study at risk of outdated data and questioning how representative the findings are of the current Fujian population. Overall, this was an important study that was well executed. I recommend that the authors address some minor issues and effect necessary revisions of the current manuscript version. Specific comments are below:

2. Examples and evidence

Major issues

N/A

Minor issues

Abstract:

1. Authors mention “blood type”. However, per the general understanding of blood type (as blood group), there are no records of data collection on blood type in the methods section, and no records of it in the results section as well. Can authors clarify what they mean by blood type and exclude it from the abstract to avoid confusion.

Introduction:

2. On page 3, paragraph 2, references 10 and 11 cited do not appear to be supportive of claim stated as authors suggest. Reference 10 talks about myopia in Europe, whereas reference 11 is on primary open-angle glaucoma with no data on myopia. Authors should re-check their references.

Materials and Methods:

3. Page 5, subsection “On-site examination”: What was the repeatability of the technician's measurement of IOP? How was intra-operator variability assessed?

4. Page 5, subsection “On-site examination”: How was the accuracy of the tonometer determined and how accurate was it? How repeatable were its readings?

5. Page 5, subsection “On-site examination”: Were outliers included/excluded in the calculation of mean IOP measurements or were they replaced with additional measurements?

6. Page 5, subsection “Refractive error assessment protocol”: How was the accuracy of the autorefractor determined and how accurate was it? How repeatable were its readings?

7. Page 5, subsection “Refractive error assessment protocol”: Were outliers included/excluded in the calculation of the arithmetic mean refractive error or were they replaced with additional measurements?

8. Page 5, subsection “Refractive error assessment protocol”: Who and how many people did the trial frame refinement? If many, how was the inter-operator variability tested and what was it?

Results:

9. On page 7-8, under the subsections “Correlations Between High Myopia and Sociodemographic Characteristics” and “Correlations Between High Myopia and Biological Characteristics”, I recommend that authors present the full tabulated results for the correlation analyses (univariate logistic regression analyses).

Discussion:

10. Page 10, paragraph 1, last line: The authors' results section seem to suggest the opposite of the claim stated here. From Table 1 and 2, higher educational level is reported to be significantly correlated with high myopia. However, the phrase “lower educational attainment” is contradictory. Can the authors clarify this?

11. Page 11, subsection “Factors Linked to High Myopia”, first sentence: Reference 24 and 26 are a duplication of the same article.

12. Page 11, subsection “Factors Linked to High Myopia”: What is the alignment between the Yugur and Han populations study and the authors' current study, considering that the authors did not assess family history of myopia, and did not find any association between high myopia and urbanization/rural-living? The only similarity between the two studies, as I can tell, is in relation to aging, which was later found not to be correlated with high myopia after adjustments in the multiple logistic regression analyses. I suggest that authors rephrase this to avoid confusion and propagating misleading information.

13. Page 11, subsection “Factors Linked to High Myopia”: What do the authors mean by certain features of educational relevance?

14. Page 12, subsection “Factors Linked to High Myopia”: The statement “Severe high myopia patients with early-onset complications (e.g., retinal detachment) may have been excluded from this community-based sample due to mobility limitations or prior hospitalization, truncating the observable risk spectrum” appears to be a limitation of the study, and therefore, is misplaced, disrupting the flow of the manuscript. Authors should consider restructuring to enhance clarity and flow.

15. Page 12, subsection “Factors Linked to High Myopia”: The sentence “The cross-sectional NHNES study suggested that myopia is linked to higher education levels in the U.S.,[29] aligning with our findings.” See comment 10.

16. Page 12, subsection “Factors Linked to High Myopia”: References 30-35 need reformatting. References 34 and 35 appear to have been included later, and so it has been numbered wrongly. Authors should reformat references to ensure appropriate and orderly numbering.

17. Page 13, “All these findings highlight context-dependent "environmental sensitivity thresholds" shaping education-myopia relationships, remind us that future research needs to comprehensively focus on the impact of multiple factors”: This statement has grammatical issues. Authors should consider rephrasing to fix grammar and enhance clarity.

18. ROC curves: Authors do not discuss figures 1 and 2 which are the ROC curves. I recommend that authors discuss what the curve means and their implications in terms of their findings.

Limitations:

19. Considering the time of the data collection, authors should address issues relating to the time-sensitivity of the results, and potential implication of outdated data.

Reviewer #2: Thank you for the opportunity to review this manuscript. The data classification and interpretation require substantial revision. I appreciate the authors’ effort in conducting this meaningful study. My comments are included as in-text comments for reference.

**********

6. PLOS authors have the option to publish the peer review history of their article (what does this mean?). If published, this will include your full peer review and any attached files.

If you choose “no”, your identity will remain anonymous but your review may still be made public.

**Do you want your identity to be public for this peer review?**  For information about this choice, including consent withdrawal, please see our Privacy Policy.

Reviewer #1: No

Reviewer #2: No

**********

[NOTE: If reviewer comments were submitted as an attachment file, they will be attached to this email and accessible via the submission site. Please log into your account, locate the manuscript record, and check for the action link "View Attachments". If this link does not appear, there are no attachment files.]

To ensure your figures meet our technical requirements, please review our figure guidelines: https://journals.plos.org/plosone/s/figures

You may also use PLOS’s free figure tool, NAAS, to help you prepare publication quality figures: https://journals.plos.org/plosone/s/figures#loc-tools-for-figure-preparation.

NAAS will assess whether your figures meet our technical requirements by comparing each figure against our figure specifications.

Attachment Submitted filename: REVIEW 1 - PONE-D-25-14251.docx

Attachment Submitted filename: renamed_595bf.docx

# Author comment: Author response to Decision Letter 1

**DOI:** 10.1371/journal.pone.0355042.r002

24 Feb 2026

Dear Reviewers,

Thank you for your thorough and insightful review of our manuscript, "Prevalence and related factors of high myopia among adults aged 50 years and older in Fujian Eye Study", Submission ID: PONE-D-25-14251. We sincerely appreciate the time and effort you have dedicated to evaluating our work and providing constructive feedback. Your comments have been invaluable in helping us improve the clarity, rigor, and overall quality of the manuscript. We have carefully considered each of your points and have made corresponding revisions to the manuscript. Our point-by-point responses are detailed below.

Reviewer 1

Minor Issues:

Abstract:

1.Comment: Authors mention “blood type”. However, per the general understanding of blood type (as blood group), there are no records of data collection on blood type in the methods section, and no records of it in the results section as well. Can authors clarify what they mean by blood type and exclude it from the abstract to avoid confusion.

Response: Thank you for catching this inconsistency. The inclusion of "blood type" in the abstract was an oversight from an earlier draft. This variable was collected in the initial questionnaire but was not included in the analysis presented in this manuscript. We have removed "blood type" and "medication use" from the Methods sentence in the Abstract. The revised sentence now reads: "Participants completed a questionnaire covering education, income, medical history, lifestyle habits (including smoking, drinking, and tea consumption)."

Introduction:

2. Comment: On page 3, paragraph 2, references 10 and 11 cited do not appear to be supportive of claim stated as authors suggest. Reference 10 talks about myopia in Europe, whereas reference 11 is on primary open-angle glaucoma with no data on myopia. Authors should re-check their references.

Response: We sincerely apologize for these citation errors. We have carefully re-checked and corrected the references.

* The claim "80-90% among young adults versus 3.6-8.2% in those over 40" is supported by references 9 (Morgan et al., 2018) and a combination of data from the corrected reference for the Tajimi Study (now cited as Ref 10, Sawada et al., 2008, which reports high myopia prevalence) and other regional studies. We have revised the reference citations Ref 11 accordingly.

* The incorrect glaucoma reference has been replaced with the appropriate citation (Sawada et al., 2008, the Tajimi Study report in Japanese) which provides the high myopia prevalence data for Japan. The reference list has been updated and renumbered consecutively.

Materials and Methods:

3. Comment: Page 5, subsection “On-site examination”: What was the repeatability of the technician's measurement of IOP? How was intra-operator variability assessed?

Response: The iCare rebound tonometer (RBT) is designed to be easy to use and exhibits good operator internal repeatability in clinical settings. The device automatically forces five consecutive measurements and obtains the average value as the final intraocular pressure value. And the personnel who measure intraocular pressure are the same operator, which reduces operational errors. We have added clarification in the revised manuscript: “The iCare tonometer has demonstrated good operator internal repeatability in field studies. Perform by the same operator to improve reliability.”

4.Comment: Page 5, subsection “On-site examination”: How was the accuracy of the tonometer determined and how accurate was it? How repeatable were its readings?

Response: The iCare TA01 tonometer used in our study has been validated with the Goldmann applanation tonometer (GAT) in multiple studies. Although there is some controversy in current reports, it cannot be denied that it still has its advantages in epidemiological investigation research and clinical practice. We added a sentence citing relevant validation literature: 'In randomized controlled and observational studies, the iCare rebound tonometer has been validated with the Goldmann flattening tonometer, showing good correlation and reproducibility.[19-21]'.

5.Comment: Page 5, subsection “On-site examination”: Were outliers included/excluded in the calculation of mean IOP measurements or were they replaced with additional measurements?

Response: The iCare device's built-in software performs an initial quality check. Measurements with poor signal quality (indicated by the device) were automatically discarded and not included in the set of five valid readings required for the average. Therefore, outliers in the form of technically flawed readings were excluded at the point of acquisition. No post-hoc statistical outlier removal was performed on the final mean IOP values used for analysis. We added a sentence “Measurements with poor signal quality (indicated by the device) were automatically discarded and not included in the set of five valid readings required for the average.” in the revised manuscript.

6.Comment: Page 5, subsection “Refractive error assessment protocol”: How was the accuracy of the autorefractor determined and how accurate was it? How repeatable were its readings?

Response: The Topcon KR800 autorefractor is a widely used, clinically validated instrument.Compared with retinoscopy, measurements obtained before and after cycloplegia with both ARs showed excellent reliability for evaluating spherical equivalent and Jackson cross-cylinder values. We have added this information: "The Topcon KR800 autorefractor is a validated instrument with high accuracy and repeatability for population-based studies."

7.Comment: Page 5, subsection “Refractive error assessment protocol”: Were outliers included/excluded in the calculation of the arithmetic mean refractive error or were they replaced with additional measurements?

Response: Similar to the IOP protocol, the three consecutive autorefractor measurements were taken under controlled conditions. If a measurement was deemed unstable or an error message appeared, the technician would retake it. The arithmetic mean was calculated from three consecutive valid measurements. No statistical outlier removal was applied afterward to these subject-level means. And we added the sentence "If a measurement was deemed unstable or an error message appeared, the technician would retake it. The arithmetic mean was calculated from three consecutive valid measurements." in the revised manuscript.

8.Comment: Page 5, subsection “Refractive error assessment protocol”: Who and how many people did the trial frame refinement? If many, how was the inter-operator variability tested and what was it?

Response: Trial frame refinement was performed by a trained and certified optometrists. To ensure consistency, the optometrist underwent standardized training using the same protocol before the study commenced. The protocol emphasized achieving the best-corrected visual acuity (BCVA) with the minimal minus sphere (or maximal plus sphere) principle. While a formal inter-operator variability assessment (e.g., Kappa statistic) was not conducted across all pairs, periodic checks and calibration sessions were held to maintain standardization. We have clarified this in the text: "BCVA was retested with optimal spherocylindrical correction determined through trial frame refinement by a trained optometrists who followed a standardized protocol."

Results:

9.  Comment: On page 7-8, under the subsections “Correlations Between High Myopia and Sociodemographic Characteristics” and “Correlations Between High Myopia and Biological Characteristics”, I recommend that authors present the full tabulated results for the correlation analyses (univariate logistic regression analyses).

Response: This is an excellent suggestion that enhances transparency. We have created a new Supplementary Table 2 titled "The univariate logistic regression of high myopia among each subgroups (n=8024)" which presents the Odds Ratio (OR), 95% Confidence Interval (CI), and P-value for every variable tested (sociodemographic, biological, and lifestyle factors). We now reference this table in the relevant Results sections: "The results of univariate logistic regression analyses for all examined factors are presented in Table 2." in the revised manuscript and original Table 2 revised as Table 3. Through the summary of the table, we have identified some writing errors and have corrected them in the manuscript. Thank you for your suggestions, which have also enabled us to discover these errors.

Discussion:

10. Comment: Page 10, paragraph 1, last line: The authors' results section seem to suggest the opposite of the claim stated here. From Table 1 and 2, higher educational level is reported to be significantly correlated with high myopia. However, the phrase “lower educational attainment” is contradictory. Can the authors clarify this?

Response: Thank you for highlighting this critical error. This was a typographical mistake. The sentence should correctly state that high myopia was associated with higher educational attainment. We have corrected it: "In this study, we identified significant correlations between high myopia and living in inland areas, higher educational attainment, and elevated intraocular pressure."

11.Comment: Page 11, subsection “Factors Linked to High Myopia”, first sentence: Reference 24 and 26 are a duplication of the same article.

Response: Thank you for your careful review. We have corrected this duplication. Reference 26 has been removed, and subsequent references have been renumbered accordingly throughout the text and in the reference list.

12.Comment: Page 11, subsection “Factors Linked to High Myopia”: What is the alignment between the Yugur and Han populations study and the authors' current study, considering that the authors did not assess family history of myopia, and did not find any association between high myopia and urbanization/rural-living? The only similarity between the two studies, as I can tell, is in relation to aging, which was later found not to be correlated with high myopia after adjustments in the multiple logistic regression analyses. I suggest that authors rephrase this to avoid confusion and propagating misleading information.

Response: We agree and thank you for this suggestion. And we have deleted this paragraph to more accurately discuss the cited study in the context of our own findings.

13.Comment: Page 11, subsection “Factors Linked to High Myopia”: What do the authors mean by certain features of educational relevance?

Response: Thank you for your careful review. This phrasing was indeed unclear. We intended to refer to factors often correlated with education in our context, such as income and prolonged near-work (proxied by phone use duration), which became non-significant in the multivariable model. We have rephrased for clarity: "Nonetheless, our study showed that high myopia was independently associated with inland residency and higher education, but not with age, height, urbanization, income, or prolonged phone use."

14.Comment: Page 12, subsection “Factors Linked to High Myopia”: The statement “Severe high myopia patients with early-onset complications (e.g., retinal detachment) may have been excluded from this community-based sample due to mobility limitations or prior hospitalization, truncating the observable risk spectrum” appears to be a limitation of the study, and therefore, is misplaced, disrupting the flow of the manuscript. Authors should consider restructuring to enhance clarity and flow.

Response: Thank you very much for your insightful advice. This point is indeed a study limitation related to potential selection bias. We have moved this sentence to the dedicated Limitations section as “Third, this study lies in its focus on the prevalence of high myopia as defined, along with an analysis of related factors such as social demographics and systemic diseases. However, it does not include a correlation analysis of eye diseases, for example, severe high myopia patients with early-onset complications (e.g., retinal detachment) may have been excluded from this community-based sample due to mobility limitations or prior hospitalization, truncating the observable risk spectrum, which we intend to address as the focal point of our future research.”.

15.Comment: Page 12, subsection “Factors Linked to High Myopia”: The sentence “The cross-sectional NHNES study suggested that myopia is linked to higher education levels in the U.S.,[29] aligning with our findings.” See comment 10.

Response: Thank you for your reminding. With the correction from Comment #10 established (our finding is higher education), this sentence is now consistent and correct as written.

16.Comment: Page 12, subsection “Factors Linked to High Myopia”: References 30-35 need reformatting. References 34 and 35 appear to have been included later, and so it has been numbered wrongly. Authors should reformat references to ensure appropriate and orderly numbering.

Response: Thank you for your reminding and we apologize for this error. The entire reference list has been carefully checked, duplicates removed (per comment 11), and all citations have been renumbered sequentially throughout the manuscript. The in-text citations and the reference list now correspond correctly.

17.Comment: Page 13, “All these findings highlight context-dependent "environmental sensitivity thresholds" shaping education-myopia relationships, remind us that future research needs to comprehensively focus on the impact of multiple factors”: This statement has grammatical issues. Authors should consider rephrasing to fix grammar and enhance clarity.

Response: Thank you for your insightful advice. We have revised the sentence for better grammar and flow: "All these findings highlight the mutual influence of multiple factors on the correlation between education and myopia, reminding us that future research needs to comprehensively investigate multiple factors and more accurately reveal the correlation between diseases and factors."

18. Comment: ROC curves: Authors do not discuss figures 1 and 2 which are the ROC curves. I recommend that authors discuss what the curve means and their implications in terms of their findings.

Response: Thank you for this observation. You are correct that our initial draft included ROC curve analysis, which indicated that IOP (AUC = 0.5740, Fig. 1) and educational background (AUC = 0.6107, Fig. 2) showed modest predictive accuracy for high myopia. However, upon further methodological review, we recognized that educational level—being a categorical variable—is not ideally suited for ROC analysis, which is typically applied to continuous or ordinal predictors. Therefore, in the final version of the manuscript, we removed the ROC results to maintain methodological rigor.

If you consider it valuable to retain or briefly mention the ROC findings in the context of IOP (a continuous variable), we would be glad to add a succinct summary in the Results section, along with a note on the interpretational limits regarding education. Please let us know your preference, and we will revise accordingly.

Fig 1. The ROC curve and AUC between high myopia and intraocular pressure.

Fig 2. The ROC curve and AUC between high myopia and educational level.

Limitations:

19. Comment: Considering the time of the data collection, authors should address issues relating to the time-sensitivity of the results, and potential implication of outdated data.

Response: This is a valid point. We have added a paragraph to the Limitations section acknowledging this: "Fourth, the data for this study were collected between 2018 and 2019. While the prevalence of high myopia in older adults is unlikely to change dramatically within a 5-7 year period, secular trends in environmental factors (e.g., digital device use) among preceding generations could influence future estimates. Nevertheless, the identified associations between high myopia and factors like education and IOP are likely to remain relevant, and this study provides a crucial baseline for this aging cohort in Southern China."

Abstract

1.Methods:

A population-based cross-sectional eye study was conducted from May 2018 to October 2019, enrolling residents aged 50 and older. Participants c

Attachment Submitted filename: 2026.01.19 Response to Reviewers.docx

# Peer review: Decision Letter 1

**DOI:** 10.1371/journal.pone.0355042.r003

**Authors:** Amithavikram Hathibelagal

4 May 2026

PONE-D-25-14251R1

Prevalence and related factors of high myopia among adults aged 50 years and older in Fujian Eye Study

PLOS One

Dear Dr. Li,

Thank you for submitting your manuscript to PLOS ONE. After careful consideration, we feel that it has merit but does not fully meet PLOS ONE’s publication criteria as it currently stands. Therefore, we invite you to submit a revised version of the manuscript that addresses the points raised during the review process.

Please submit your revised manuscript by Jun 18 2026 11:59PM. If you will need more time than this to complete your revisions, please reply to this message or contact the journal office at plosone@plos.org. When you're ready to submit your revision, log on to https://www.editorialmanager.com/pone/ and select the 'Submissions Needing Revision' folder to locate your manuscript file.

Please include the following items when submitting your revised manuscript:

- A letter that responds to each point raised by the academic editor and reviewer(s). You should upload this letter as a separate file labeled 'Response to Reviewers'.
- A marked-up copy of your manuscript that highlights changes made to the original version. You should upload this as a separate file labeled 'Revised Manuscript with Track Changes'.
- An unmarked version of your revised paper without tracked changes. You should upload this as a separate file labeled 'Manuscript'.

If you would like to make changes to your financial disclosure, please include your updated statement in your cover letter. Guidelines for resubmitting your figure files are available below the reviewer comments at the end of this letter.

If applicable, we recommend that you deposit your laboratory protocols in protocols.io to enhance the reproducibility of your results. Protocols.io assigns your protocol its own identifier (DOI) so that it can be cited independently in the future. For instructions see: https://journals.plos.org/plosone/s/submission-guidelines#loc-laboratory-protocols. Additionally, PLOS ONE offers an option for publishing peer-reviewed Lab Protocol articles, which describe protocols hosted on protocols.io. Read more information on sharing protocols at https://plos.org/protocols?utm_medium=editorial-email&utm_source=authorletters&utm_campaign=protocols.

As the corresponding author, your ORCID iD is verified in the submission system and will appear in the published article. PLOS supports the use of ORCID, and we encourage all coauthors to register for an ORCID iD and use it as well. Please encourage your coauthors to verify their ORCID iD within the submission system before final acceptance, as unverified ORCID iDs will not appear in the published article. *Only*  the individual author can complete the verification step; PLOS staff *cannot*  verify ORCID iDs on behalf of authors.

We look forward to receiving your revised manuscript.

Kind regards,

Amithavikram R Hathibelagal, Ph.D.

Academic Editor

PLOS One

Journal Requirements:

If the reviewer comments include a recommendation to cite specific previously published works, please review and evaluate these publications to determine whether they are relevant and should be cited. There is no requirement to cite these works unless the editor has indicated otherwise.

Please review your reference list to ensure that it is complete and correct. If you have cited papers that have been retracted, please include the rationale for doing so in the manuscript text, or remove these references and replace them with relevant current references. Any changes to the reference list should be mentioned in the rebuttal letter that accompanies your revised manuscript. If you need to cite a retracted article, indicate the article’s retracted status in the References list and also include a citation and full reference for the retraction notice.

Additional Editor Comments (if provided):

Please address the additional comments

[Note: HTML markup is below. Please do not edit.]

Reviewers' comments:

Reviewer's Responses to Questions

**Comments to the Author**

1. If the authors have adequately addressed your comments raised in a previous round of review and you feel that this manuscript is now acceptable for publication, you may indicate that here to bypass the “Comments to the Author” section, enter your conflict of interest statement in the “Confidential to Editor” section, and submit your "Accept" recommendation.

Reviewer #1: All comments have been addressed

Reviewer #2: All comments have been addressed

**********

2. Is the manuscript technically sound, and do the data support the conclusions?

The manuscript must describe a technically sound piece of scientific research with data that supports the conclusions. Experiments must have been conducted rigorously, with appropriate controls, replication, and sample sizes. The conclusions must be drawn appropriately based on the data presented.

Reviewer #1: Yes

Reviewer #2: Yes

**********

3. Has the statistical analysis been performed appropriately and rigorously?

Reviewer #1: Yes

Reviewer #2: Yes

**********

4. Have the authors made all data underlying the findings in their manuscript fully available?

The PLOS Data policy requires authors to make all data underlying the findings described in their manuscript fully available without restriction, with rare exception (please refer to the Data Availability Statement in the manuscript PDF file). The data should be provided as part of the manuscript or its supporting information, or deposited to a public repository. For example, in addition to summary statistics, the data points behind means, medians and variance measures should be available. If there are restrictions on publicly sharing data—e.g. participant privacy or use of data from a third party—those must be specified.

Reviewer #1: (No Response)

Reviewer #2: Yes

**********

5. Is the manuscript presented in an intelligible fashion and written in standard English?

PLOS ONE does not copyedit accepted manuscripts, so the language in submitted articles must be clear, correct, and unambiguous. Any typographical or grammatical errors should be corrected at revision, so please note any specific errors here.

Reviewer #1: No

Reviewer #2: Yes

**********

6. Review Comments to the Author

Please use the space provided to explain your answers to the questions above. You may also include additional comments for the author, including concerns about dual publication, research ethics, or publication ethics. (Please upload your review as an attachment if it exceeds 20,000 characters)

Reviewer #1: The authors have addressed all the comments and concerns raised in the previous review. However, there are a few grammatical errors that impedes clarity and understanding of the manuscript. The authors are advised to employ the services of an experienced English writer to correct all errors and enhance the flow and clarity of the manuscript.

Again, the authors should cross-check every reference cited to make sure it is well numbered and correlated with the claims and statements made throughout the manuscript, since references have been updated.

Few specific instances of grammatical errors are cited below:

Minor issues

Abstract:

1. “Participants completed a questionnaire covering education, income, medical history, lifestyle habits (including smoking, drinking, and tea consumption).” Consider inserting an “and” between the last two items on the list: “……medical history, and lifestyle habits (including……”.

Materials and Methods:

2. “The iCare tonometer has demonstrated good operator internal repeatability in field studies. Perform by the same operator to improve reliability.” The second clause appears to stand alone which makes it incomplete.

3. "BCVA was retested with optimal spherocylindrical correction determined through trial frame refinement by a trained optometrists who followed a standardized protocol." Was it a single trained optometrist or multiple optometrists? It is not quite clear from the use of “a” and plural form “optometrists”.

Reviewer #2: The authors’ response was as follows:

“Thank you for this important point regarding the direction of the association between high myopia and elevated intraocular pressure (IOP). We fully agree that clarifying the potential causal pathway is of significant interest. In the revised Discussion section, we have added a dedicated paragraph addressing this issue. We explicitly state that, ‘As a cross-sectional study, our findings can demonstrate a statistical association but cannot establish causality or determine the direction of the relationship. Recent a review showed anatomically, thinner trabecular meshwork increases the risk of high IOP, conversely lamina cribrosa defects may offer a fluid outlet, potentially mitigating the pressure.[38] While another review showed that scleral matrix remodeling has been shown to contribute to the biomechanical susceptibility of the sclera to accommodation-induced IOP fluctuations, resulting in reduced scleral thickness, axial length (AL) elongation, and axial myopia.[39] Thus, longitudinal or interventional studies are needed to clarify any clarified causal relationship in the future.’ This limitation of the cross-sectional design is already acknowledged in the ‘Limitations’ subsection of the Discussion.”

In response, I would like to provide the following additional comments. While the authors have attempted to address the reviewer’s concern, the revised Discussion remains largely descriptive and does not sufficiently clarify the potential directionality of the relationship between high myopia and elevated IOP. Simply stating that causality cannot be determined in a cross-sectional design is appropriate but does not fully resolve the issue raised.

The authors are encouraged to further elaborate on which direction of association is more strongly supported by current evidence (e.g., high myopia leading to increased susceptibility to IOP-related damage vs. elevated IOP contributing to axial elongation), and to provide a more critical and integrative interpretation of the cited literature rather than listing possible mechanisms.

In addition, references [38] and [39], which are cited in the revised Discussion, do not appear to be included in the reference list. The authors should verify and ensure consistency between in-text citations and the reference list.

Furthermore, the statements regarding IOP in the Limitations section would benefit from more comprehensive and appropriate citation of the existing literature to support the claims.

Overall, the current response remains insufficient to fully address the reviewer’s original concern, and further clarification and revision are warranted.

**********

7. PLOS authors have the option to publish the peer review history of their article (what does this mean?). If published, this will include your full peer review and any attached files.

If you choose “no”, your identity will remain anonymous but your review may still be made public.

**Do you want your identity to be public for this peer review?**  For information about this choice, including consent withdrawal, please see our Privacy Policy.

Reviewer #1: No

Reviewer #2: No

**********

[NOTE: If reviewer comments were submitted as an attachment file, they will be attached to this email and accessible via the submission site. Please log into your account, locate the manuscript record, and check for the action link "View Attachments". If this link does not appear, there are no attachment files.]

To ensure your figures meet our technical requirements, please review our figure guidelines: https://journals.plos.org/plosone/s/figures

You may also use PLOS’s free figure tool, NAAS, to help you prepare publication quality figures: https://journals.plos.org/plosone/s/figures#loc-tools-for-figure-preparation.

NAAS will assess whether your figures meet our technical requirements by comparing each figure against our figure specifications.

Attachment Submitted filename: REVIEW 1 - PONE-D-25-14251_R1.docx

# Author comment: Author response to Decision Letter 2

**DOI:** 10.1371/journal.pone.0355042.r004

12 Jun 2026

Dear Reviewer,

We sincerely thank you for your careful review and constructive comments on our manuscript. We have carefully considered all the points you raised and have revised the whole manuscript according to your advice. Below we provide our point-by-point responses to your comments.

Comment 1 (Abstract):

“Participants completed a questionnaire covering education, income, medical history, lifestyle habits (including smoking, drinking, and tea consumption).” Consider inserting an “and” between the last two items on the list.

Response: Thank you for this suggestion. We have revised the sentence as recommended. The corrected sentence now reads:

“Participants completed a questionnaire covering education, income, medical history, and lifestyle habits (including smoking, drinking, and tea consumption).”

The change has been made in the revised manuscript (see Abstract section).

Comment 2 (Materials and Methods):

“The iCare tonometer has demonstrated good operator internal repeatability in field studies. Perform by the same operator to improve reliability.” The second clause appears to stand alone which makes it incomplete.

Response: We agree with your observation. The original sentence was incomplete. We have rewritten it for clarity and grammatical completeness. The revised sentence now reads:

“The iCare tonometer has demonstrated good intra-operator repeatability in field studies. In order to improve reliability, our study employed the same operator for all measurements.”

This change has been incorporated into the Materials and Methods section of the revised manuscript.

Comment 3 (Materials and Methods):

“BCVA was retested with optimal spherocylindrical correction determined through trial frame refinement by a trained optometrists who followed a standardized protocol.” Was it a single trained optometrist or multiple optometrists? It is not quite clear from the use of “a” and plural form “optometrists”.

Response: Thank you for pointing this out. We carefully rechecked the entire manuscript, and we were unable to locate the exact sentence you quoted. We believe that this sentence had already been corrected or removed in an earlier revision prior to submission. In the current version of our manuscript, there is no ambiguous wording regarding “a trained optometrists.” All descriptions of the optometrist(s) have been clarified, specifying that a single trained optometrist performed all BCVA measurements following a standardized protocol. Should any trace of the previous wording have inadvertently remained, we have now thoroughly reviewed and ensured consistency throughout the text.

Again, we greatly appreciate your thorough and insightful review. We believe that the revised manuscript has been significantly improved in terms of language clarity and scientific rigor. Thank you for your continued guidance.

Sincerely,

Yang Li

On behalf of all authors

# Peer review: Decision Letter 2

**DOI:** 10.1371/journal.pone.0355042.r005

**Authors:** Rajiv Mohan

17 Jul 2026

Prevalence and related factors of high myopia among adults aged 50 years and older in Fujian Eye Study

PONE-D-25-14251R2

Dear Dr. Li,

We’re pleased to inform you that your manuscript has been judged scientifically suitable for publication and will be formally accepted for publication once it meets all outstanding technical requirements.

Within one week, you’ll receive an e-mail detailing the required amendments. When these have been addressed, you’ll receive a formal acceptance letter and your manuscript will be scheduled for publication.

An invoice will be generated when your article is formally accepted. Please note, if your institution has a publishing partnership with PLOS and your article meets the relevant criteria, all or part of your publication costs will be covered. Please make sure your user information is up-to-date by logging into Editorial Manager at Editorial Manager® and clicking the ‘Update My Information' link at the top of the page. For questions related to billing, please contact billing support.

If your institution or institutions have a press office, please notify them about your upcoming paper to help maximize its impact. If they’ll be preparing press materials, please inform our press team as soon as possible -- no later than 48 hours after receiving the formal acceptance. Your manuscript will remain under strict press embargo until 2 pm Eastern Time on the date of publication. For more information, please contact onepress@plos.org.

Kind regards,

Rajiv R. Mohan, Ph.D.

Academic Editor

PLOS One

Additional Editor Comments (optional):

Dear authors,

I am happy to inform that your revisions/changes adequately addressed all concerns. The manuscript is accepted for publication. Congratulations!

Rajiv Mohan

Reviewers' comments:

Reviewer's Responses to Questions

**Comments to the Author**

1. If the authors have adequately addressed your comments raised in a previous round of review and you feel that this manuscript is now acceptable for publication, you may indicate that here to bypass the “Comments to the Author” section, enter your conflict of interest statement in the “Confidential to Editor” section, and submit your "Accept" recommendation.

Reviewer #1: All comments have been addressed

Reviewer #2: All comments have been addressed

**********

2. Is the manuscript technically sound, and do the data support the conclusions?

The manuscript must describe a technically sound piece of scientific research with data that supports the conclusions. Experiments must have been conducted rigorously, with appropriate controls, replication, and sample sizes. The conclusions must be drawn appropriately based on the data presented.

Reviewer #1: Yes

Reviewer #2: Yes

**********

3. Has the statistical analysis been performed appropriately and rigorously?

Reviewer #1: Yes

Reviewer #2: Yes

**********

4. Have the authors made all data underlying the findings in their manuscript fully available?

The PLOS Data policy requires authors to make all data underlying the findings described in their manuscript fully available without restriction, with rare exception (please refer to the Data Availability Statement in the manuscript PDF file). The data should be provided as part of the manuscript or its supporting information, or deposited to a public repository. For example, in addition to summary statistics, the data points behind means, medians and variance measures should be available. If there are restrictions on publicly sharing data—e.g. participant privacy or use of data from a third party—those must be specified.

Reviewer #1: (No Response)

Reviewer #2: Yes

**********

5. Is the manuscript presented in an intelligible fashion and written in standard English?

PLOS ONE does not copyedit accepted manuscripts, so the language in submitted articles must be clear, correct, and unambiguous. Any typographical or grammatical errors should be corrected at revision, so please note any specific errors here.

Reviewer #1: Yes

Reviewer #2: Yes

**********

6. Review Comments to the Author

Please use the space provided to explain your answers to the questions above. You may also include additional comments for the author, including concerns about dual publication, research ethics, or publication ethics. (Please upload your review as an attachment if it exceeds 20,000 characters)

Reviewer #1: The authors have addressed all my queries and concerns, and this has improved on the overall quality and clarity of the manuscript.

Thank you.

Reviewer #2: Thank you for having me a great opportunity to review this article.

All comments have been addressed

**********

7. PLOS authors have the option to publish the peer review history of their article (what does this mean?). If published, this will include your full peer review and any attached files.

If you choose “no”, your identity will remain anonymous but your review may still be made public.

**Do you want your identity to be public for this peer review?**  For information about this choice, including consent withdrawal, please see our Privacy Policy.

Reviewer #1: No

Reviewer #2: No

**********

# Editor report: Acceptance letter

**DOI:** 10.1371/journal.pone.0355042.r006

**Authors:** Rajiv Mohan

PONE-D-25-14251R2

PLOS One

Dear Dr. Li,

I'm pleased to inform you that your manuscript has been deemed suitable for publication in PLOS One. Congratulations! Your manuscript is now being handed over to our production team.

At this stage, our production department will prepare your paper for publication. This includes ensuring the following:

* All references, tables, and figures are properly cited

* All relevant supporting information is included in the manuscript submission,

* There are no issues that prevent the paper from being properly typeset

You will receive further instructions from the production team, including instructions on how to review your proof when it is ready. Please keep in mind that we are working through a large volume of accepted articles, so please give us a few days to review your paper and let you know the next and final steps.

Lastly, if your institution or institutions have a press office, please let them know about your upcoming paper now to help maximize its impact. If they'll be preparing press materials, please inform our press team within the next 48 hours. Your manuscript will remain under strict press embargo until 2 pm Eastern Time on the date of publication. For more information, please contact onepress@plos.org.

You will receive an invoice from PLOS for your publication fee after your manuscript has reached the completed accept phase. If you receive an email requesting payment before acceptance or for any other service, this may be a phishing scheme. Learn how to identify phishing emails and protect your accounts at https://explore.plos.org/phishing.

If we can help with anything else, please email us at customercare@plos.org.

Thank you for submitting your work to PLOS ONE and supporting open access.

Kind regards,

PLOS ONE Editorial Office Staff

on behalf of

Dr. Rajiv R. Mohan

Academic Editor

PLOS One
